# The Critical Role of Head Movements for Spatial Representation During Bumblebees Learning Flight

**DOI:** 10.3389/fnbeh.2020.606590

**Published:** 2021-01-19

**Authors:** Charlotte Doussot, Olivier J. N. Bertrand, Martin Egelhaaf

**Affiliations:** Department of Neurobiology, University of Bielefeld, Bielefeld, Germany

**Keywords:** active vision, hymenopterans, navigation, view-matching, optic-flow, visual homing, motion-parallax, visual learning

## Abstract

Bumblebees perform complex flight maneuvers around the barely visible entrance of their nest upon their first departures. During these flights bees learn visual information about the surroundings, possibly including its spatial layout. They rely on this information to return home. Depth information can be derived from the apparent motion of the scenery on the bees' retina. This motion is shaped by the animal's flight and orientation: Bees employ a saccadic flight and gaze strategy, where rapid turns of the head (saccades) alternate with flight segments of apparently constant gaze direction (intersaccades). When during intersaccades the gaze direction is kept relatively constant, the apparent motion contains information about the distance of the animal to environmental objects, and thus, in an egocentric reference frame. Alternatively, when the gaze direction rotates around a fixed point in space, the animal perceives the depth structure relative to this pivot point, i.e., in an allocentric reference frame. If the pivot point is at the nest-hole, the information is nest-centric. Here, we investigate in which reference frames bumblebees perceive depth information during their learning flights. By precisely tracking the head orientation, we found that half of the time, the head appears to pivot actively. However, only few of the corresponding pivot points are close to the nest entrance. Our results indicate that bumblebees perceive visual information in several reference frames when they learn about the surroundings of a behaviorally relevant location.

## 1. Introduction

When describing a location of an object in its environment, we often narrate about its relationships to other items; for example, the mountain is on the right of the river. This description is based on an environmental representation from an allocentric perspective, i.e., it is the relation between the different objects which matters without explicit reference to the observer. However, while moving through this environment, the observer establishes from an egocentric perspective, a relation between the self and the objects in the environment. Both types of representations have been concluded to co-exist in the human brain (Burgess, 2006; Mou et al., 2006; Avraamides and Kelly, 2008). However, not only humans but many other navigating animals, even the ones with tiny brains, such as insects, are confronted with the problem of spatial representation (Wehner et al., 1996).

For example, bees and ants, commute daily between their underground nest and a food source to ensure the colony's survival. To return home, these insects have been shown to use a variety of strategies. For example, they can use path integration (PI) and view matching (Wehner, 2009; Zeil, 2012; Webb, 2019; Sun et al., 2020). PI gives them knowledge about their position relative to their nest-hole (Muller and Wehner, 1988; Wehner et al., 1996). However, this egocentered information might be uncertain in the nest vicinity (Cheung et al., 2007; Cheung and Vickerstaff, 2010; Wystrach et al., 2015; Hoinville and Wehner, 2018; Webb, 2019). Therefore, insects also rely on view-matching in the final approach to their nest (Zeil, 2012; Webb, 2019; Sun et al., 2020). An insect is thought to compare its current retinotopic representation of the environment with one or several previously-stored views of its nest surroundings (Doussot et al., 2020a). These views might be learned during its early trips out of the nest, when naïve to the visual surroundings. Naive bumblebees perform convoluted maneuvers around their nest mostly looking back at it. These peculiar flight sequences have been interpreted as learning flights (Philippides et al., 2013; Degen et al., 2018; Lobecke et al., 2018; Robert et al., 2018), and are assumed to be the result of an intrinsic learning routine taking place while exiting the nest for the first times and at flower sites. The thereby collected images, encoding the brightness and colors of the panorama, is one way to represent the nest's visual surroundings. However, insects like bees may use views containing depth information. Indeed brightness information alone cannot account for homing behavior under all observed environmental conditions (Zeil, 1993b; Fry and Wehner, 2002; Vardy and Möller, 2005; Dittmar et al., 2010, 2011; Riabinina et al., 2014; Boeddeker et al., 2015; Collett and Zeil, 2018; Lobecke et al., 2018; Doussot et al., 2020a). Irrespective of the stored information about the environment, it is still unknown where exactly this information is gathered during the learning flights and in which frame of reference, be it allocentric or egocentric, it is represented in the insect brain.

Distance information is available to bees while flying, and therefore, also during learning flights, thanks to a saccadic and gaze strategy. This saccadic flight strategy is observed in many flying animal [blowfly: (Van Hateren and Schilstra, 1999), fruit-flies (Mronz and Lehmann, 2008), honeybees: (Boeddeker et al., 2010), hoverflies: (Geurten et al., 2010), birds (Eckmeier et al., 2008; Ros and Biewener, 2017)]. On a fine time scale, the bee stabilizes its head for some time (intersaccades) and interrupts this apparent stabilization only when performing brief high-velocity turns around its vertical axis (saccades). Flying insects are usually thought to perform this active behavior to segregate between rotational movements and translational ones (Van Hateren and Schilstra, 1999; Boeddeker et al., 2010; Egelhaaf et al., 2012; Kern et al., 2012; Serres and Ruffier, 2017). Indeed, during pure rotations, the amplitude of image displacements (i.e., the optic flow) perceived by the eyes is independent of the spatial arrangement of objects. Since the eye is fixed to the head capsule in insects, rotational movement of the head does not carry information about the distance to objects and depends only on the animal's head motion. In contrast, when the head direction is kept stable, depth information can be obtained from the pattern of apparent motion since it is dominated by translational optic flow (“motion-parallax”). The closer an object is to the observer, the quicker its displacements on the retina will be. In that respect, the spatial representation is based on an egocentric reference frame (i.e., centered at the observer). Hence, bumblebees may well be able to infer their distance to surrounding objects during the short intersaccadic intervals.

However, when investigating head movements of bumblebees during learning flights (Riabinina et al., 2014) and Boeddeker et al. (2015) suggested that, apart from noise, there may be systematic residual slow-velocity head rotations along the vertical axis during intersaccades, inducing a pivoting-parallax. Such rotations might have a direct impact on the reference frame in which spatial environmental information is represented. Indeed, while rotating, the depth information available from the optic-flow (OF) pattern is linked to the point around which these rotations are performed, i.e., the pivot point. Riabinina et al. (2014) suggested that during certain intersaccades bumblebees perform a pivoting-parallax centered around the nest [as was previously indicated for wasps' learning flights (Zeil, 1993a,b)]. However, a pivoting-parallax may not necessarily be centered around the nest entrance, and may consequently, correspond to an allocentric reference frame different to a nest-centric one.

The reference frame in which bees are perceiving the spatial layout of the environment, be it egocentric, allocentric or nest-centric, can be evaluated from the head orientation of bumblebees during intersaccades while performing learning flights. To meet the methodological challenge imposed by head tracking in the context of free flights and to reduce the possibility of methodological noise and systematic inaccuracies, we tracked at a high precision the head orientation of bumblebees, *Bombus terrrestris*, during the beginning of their learning flights, i.e., close to their nest entrance. The performance and reliability of our method are assessed systematically. On this basis, we characterized the head kinematics around all three axes of rotation (x, y, and z-axis). First, we describe in detail the head kinematics during learning flights and specifically scrutinize the presence of rotations during the intersaccadic intervals. Second, on the basis of the determined head rotations and a signal-to-noise-ratio analysis, we assign to each intersaccade the most likely active vision strategy which is adopted, i.e., pivoting or motion-parallax. Importantly, we do not make any assumptions in this analysis around which point in space the animal may pivot. Therefore, the pivot points' locations are estimated for the intersaccadic intervals with significant residual overall rotations. We discuss the implications of these results for the reference-frame in which views might be collected during learning flights and the potential impact for visual homing by view-matching.

## 2. Materials and Methods

### 2.1. Experimental Set-Up

**Figure 1:**
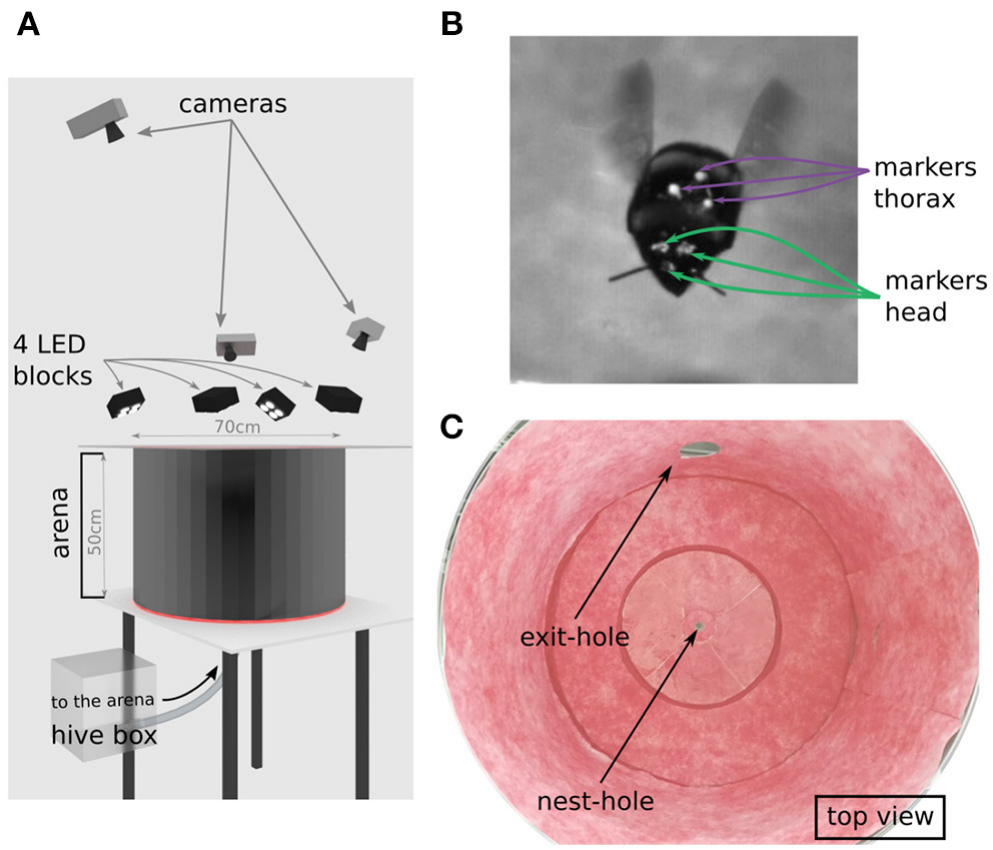
Experimental set-up. **(A)** Representation of the experimental set-up recreated with the software Blender. The bumblebee enters the flight arena through the nest-hole connected by a tube to the hive. The bumblebee takes off from the center of the arena. Learning flights were recorded by three cameras from above the arena. The flight arena was illuminated by four blocks of four LEDs. The roof of the arena was a transparent acrylic plate. **(B)** Single cropped frame from our footage showing a marked bumblebee during a learning flight; green arrows indicate the head markers and purple arrows point to the three thorax markers. **(C)** Photograph of the inside texture of the arena as used during experiments, showing nest-hole and exit-hole to the foraging. Walls are covered with a red noise pattern.

We used a hive of *Bombus terrestris* with a small number of individuals provided by Koppert B.V., The Netherlands. Bumblebees had access to pollen *ad libitum* in their hive. The hive was placed in a perforated acrylic box, allowing for enough ventilation, connected by transparent tubing to a flight arena (Figure 1A). The bumblebees entered the metallic cylindrical flight arena (diameter: 70 cm, height: 50 cm) through a 1 cm hole in the middle of its floor. To allow the lighting of the set-up and recording of learning flights, the arena was covered with a transparent acrylic lid. The walls and floor of the arena were covered by a random white and red pattern that was spatially low-pass filtered leading to a pattern with a 1/f frequency distribution (pink noise) providing the bumblebees with enough contrast to use the OF (Figure 1C). We did not introduce artificial landmarks, in the hope that the head movements will not be biased by the animal looking at prominent landmarks in the environment. Indeed, such landmarks might drive the attention of the bumblebees during learning (Nicholson et al., 1999; Baddeley et al., 2009), thus, impairing the investigation about a potential pivoting-parallax performed around the nest-hole. After completing their learning flights, bumblebees were able to leave the flight arena via a hole of 10 cm diameter, the exit-hole, giving access to a transparent tube leading to a foraging chamber with an artificial feeder containing a sucrose solution (30% saccharose). However, during our experiments no bumblebee with markers entered the foraging chamber. This exit-hole of 10 cm diameter in addition to the nest-hole itself, was the only distinct visual landmark which was in the height range where bumblebee would fly in our recordings (exit-hole center located at 12 cm above the ground).

### 2.2. Animal Preparation

Before connecting the hive to the experimental set-up, we caught several bumblebees from the hive to place head and thorax markers on them. The bees were kept under mechanical constraint in a custom made marking tool adapted to bumblebee size, to be marked without anesthesia. We drew three small dots (~1 mm diameter each) of acrylic paint on the bee's head: one above each eye and the third between the two eyes at the height of the antennae scape insertion point, similarly to Riabinina et al. (2014). During the marking procedure, attention was paid not to cover the ocelli and the compound eyes (Figure 1B). We used an equilateral triangle (side length of 5 mm) of black paper with a white pearl dot (1 mm diameter) at each apex to mark the thorax (inspired from Ravi et al., 2013). These 3D thorax markers were fixed with a mixture of bee wax and tree sap centered between the two wings and in alignment with the longitudinal body axis. After marking, we placed the bumblebees back to the hive. To assess potential individual differences or an experience-dependent impact on the head dynamics during the initial phase of learning flights, we post-identified the different individuals. A tag for identification could not be placed on the bumblebees as they would interfere with the automatic tracking of our markers. From a close look on the recordings, the tiny differences between the head markers' shape were used for identification of the bumblebee. We conclude that four flights are performed by the individual “a” (flights id numbers 1, 2, 3, 4 in chronological order), and flight 5 and 6 being learning flights of two different individuals “b” and “c,” thus; leading to three individuals and six recordings.

### 2.3. Tracking of Head and Thorax Markers

We recorded the flights via three high-speed cameras (Optronis CR3000x2) with a resolution of 1, 710 × 1, 696 pixels. The three cameras placed above the arena at different positions and viewing directions, recorded a volume of ~ 10 × 10 × 10 cm^3^ around the nest-hole (Figure 1A). The recording area was restricted to just a small part of the arena to allow monitoring the head and thorax orientation at a sufficiently high resolution. The recording volume was illuminated by four blocks of four LEDs each (HIB Multihead LED, HS vision GmbH, Germany) (Figure 1A). When a bee entered the arena from the nest-hole, we started the recording as soon as the bumblebee took-off to perform a learning flight. Recordings were made at a shutter speed of 1/2, 000 s, a frame rate of 500 frames per second, and for ~11 s. The three cameras were calibrated with the Matlab toolbox dltdv5 (Hedrick, 2008). We tracked the head and thorax markers with a custom-made Python script, based on OpenCV. The videos were then manually reviewed with the software IVtrace (https://opensource.cit-ec.de/projects/ivtools); in case of tracking errors, the marker positions could be manually set. Finally, the markers' positions in 3D space were reconstructed (Hedrick, 2008). After each learning flight, the recorded bumblebee returned to the hive without visiting the foraging chamber. This finding is likely to be due to the experiments being performed only over 5 days; so most bumblebees did not have enough time to learn the location of the foraging chamber. Besides, the food stock in the hive was partly filled with honey upon delivery. In this manner, our recorded bumblebees (a, b, and c) could be considered as “novices” without the urgent need to collect food. For example, ant novices are thought to perform learning walks of a similar structure until they accidentally discover a food source (Fleischmann, 2019). This observation, extended to our bumblebees, allows us to consider the multiple learning flights performed by individual “a” in a similar way.

### 2.4. Head and Thorax Spatial Orientation

**Figure 2:**
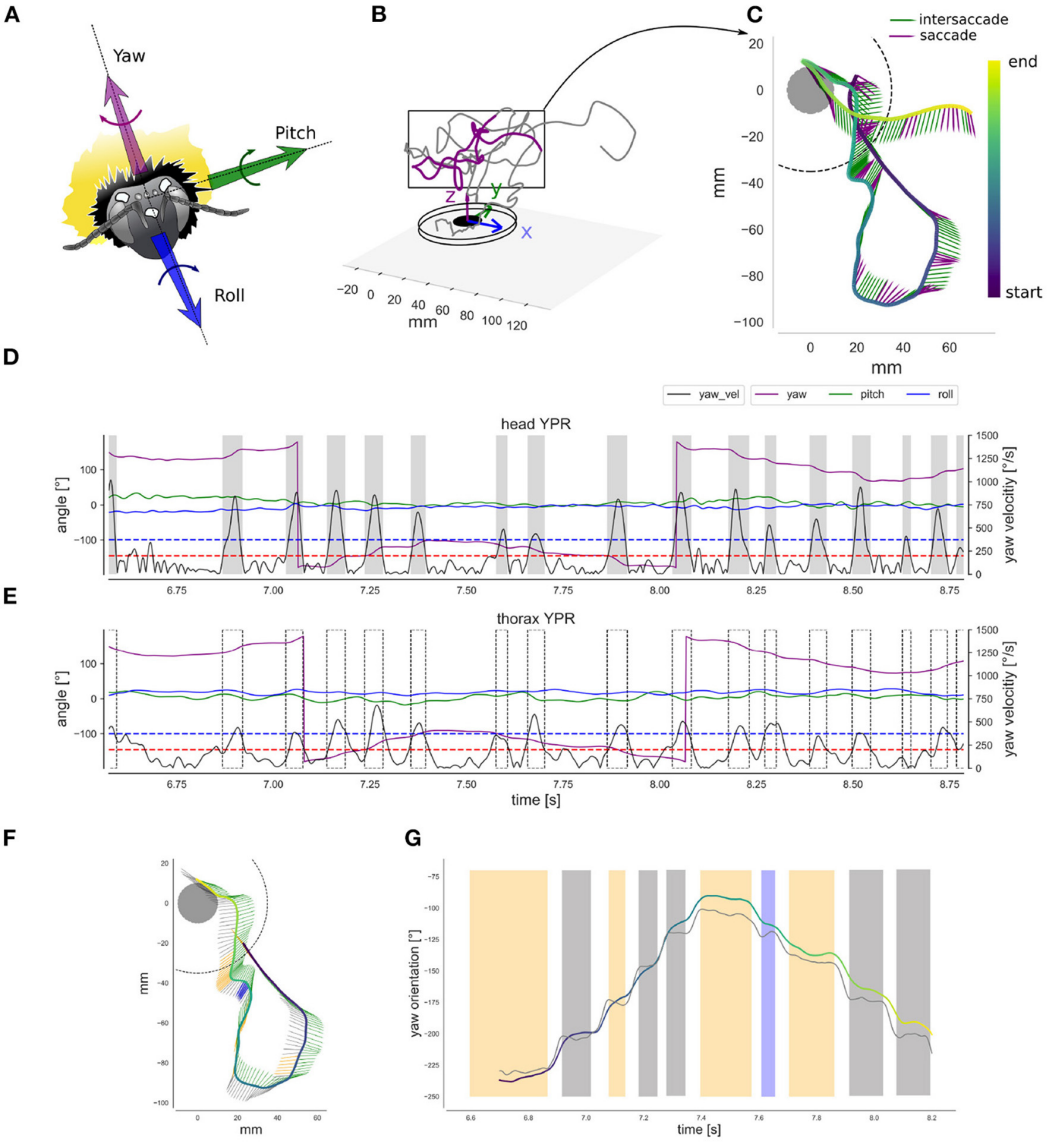
Head and thorax spatial orientation **(A)** The head coordinate-system: the bumblebee head with the three markers and the yaw, pitch and roll axis. **(B)** The world coordinate system: 3D representation of a learning flight's initial phase. **(C)** Top-view of the learning flight section showing the down-sampled yaw orientation. The head direction is indicated by the arrow's head. Time along the trajectory is indicated by the arrow head color, following the “color bar” at the right. Purple arrows indicate saccades and green arrows intersaccades. **(D)** Filtered time courses of the head YPR orientation for the flight section shown in **(C)**, with yaw in purple, pitch in green, roll in blue. Each orientation is overlaid with the standard deviation of the error in degrees (too small to be visible). Rectified yaw velocity on the right axis (black). Gray shaded areas represents saccades determined by the two-thresholds method (see text): for the head, onset threshold (upper blue line) = 372.42°· s^−1^ and ending threshold 2 (lower red line) = 200.5°· s^−1^. **(E)** Filtered time courses of the thorax YPR orientation (left-axis) and yaw-rectified velocity (right-axis) for the flight section shown in **(C)**, similar legends as in **(D)**. Saccades as defined on the basis of head velocity is indicated by dotted blocks. **(F)** Top-view of a smaller learning flight section showing the down-sampled yaw orientation for the head and the thorax. The head direction is indicated by the arrow's tail in gray. The thorax direction by the arrow's head (green arrows). Time along the trajectory is indicated by the arrows head color. Time colored indication is similar to **(E)**. Blue arrows indicate a positive head yaw drift above 5° and the orange a negative drift below 5°. **(G)** Filtered time courses of the head (gray line) and thorax yaw orientation (colored line). The blue and orange colors indicates strong drift as in **(F)**, and gray indicates intersaccades with less drift. The thorax orientation is plotted with a colored line. Its color indicates time, and is the same as in **(F)**. Head and thorax orientation are not aligned in the second half of the section, so the head is orientated rightward relative to the thorax.

To accurately monitor the movements and orientation of the animal's thorax and head, we reconstructed the head and thorax markers' positions. Head and thorax orientation are subject to different functional constraints and often not aligned with each other. For instance, the thorax executes large roll movements during curves or sideways translation to generate appropriate torque moments or forces, while the head compensates for these thorax rotations largely (Boeddeker and Hemmi, 2010; Boeddeker et al., 2015). Therefore, head orientation and gaze direction can be inferred to a relatively limited extent from the thorax orientation (Odenthal et al., 2020). To reconstruct the head and thorax orientation we defined three coordinate systems: the reference frame of (1) the head- (HCS) and (2) the thorax-centered (TCS) coordinate systems as defined by the head or thorax markers, respectively, and (3) the world coordinate system (WCS) attached to the flight arena (Figures 2A,B). The global head and thorax orientations were determined as the angle required to align the HCS and TCS, respectively, with the world coordinate system. We computed for each captured frame the instantaneous yaw, pitch, and roll (YPR) angles of head and thorax, respectively. Each angle was determined in the following order corresponding to Diebel's convention (Diebel, 2006): first rotation along with the animal's roll (x-axis); second, rotation along the pitch axis (y-axis); third, rotation along the yaw axis (z-axis). Similar methods have already been used to estimate instantaneous orientations in previous studies on a variety of flying animals (Pete et al., 2015; Ravi et al., 2015). Each YPR angle was smoothed with a planar cubic spline function (Scipy.signal package) with smoothing parameter (λ = 150) interpreted as the degree of freedom, and estimated by generalized cross-validation criterion (smooth.spline R function). Cubic splines are often used in biomechanics data filtering (Woltring, 1985). Examples of filtered time courses of the head and thorax YPR angles are shown in Figure 2. Finally, from the YPR orientation, the respective angular velocities referred as *w*_*x*_, *w*_*y*_, and *w*_*z*_ were expressed in the HCS and TCS, following Diebels et al. (Equations 39 and 40, p. 9), so velocities are defined along the x, y, and z-axis of the corresponding body segment (Diebel, 2006).

### 2.5. Saccade Extraction

Based on the rectified yaw velocity time courses (obtained from smoothed yaw angle time courses), we investigated the saccadic flight and gaze strategy. To do so, we determined head saccades as periods where yaw velocity exceeded a given threshold (372.42°· s^−1^, as in Riabinina et al., 2014) and until it decreased below another threshold (200.53°· s^−1^) (Figure 2). We applied this two-thresholds method automatically to all flights (Figure 2D).

### 2.6. Tracking Error Propagation

While tracking a point in space, there exists a certain positional error in the 3D space. If several points are tracked, and a solid is attached to these, the resulting orientation of this solid is influenced by the positional error given for the different points. Therefore, we needed to propagate the positional error of the markers induced by our tracking to the head YPR orientation. This was done at each time point and for each trajectory. To estimate our tracking error, we calculated at each time instance *t*, the deviation from the average distance between each pair of markers across all flight. This average distance is hypothesized to be the actual distance between the markers, which is constant for each pair of markers. Therefore, any deviation from this value is the result of a tracking error. We kept the worst “tracking error,” ϵ(*t*), from all the pairs. For simplification, this error was assumed to be the same for each marker and along the different axes. Consequently, we populated a co-variance matrix, σ_*measured*_ (Equation 1) with the “co-variance” components not being considered, i.e., the off-diagonals terms are 0, since the errors of the different measures are considered uncorrelated (Hugues and Hase, 2010) (see Equation 1).

(1) $$\begin{array}{l} \;\begin{array}{ccc} x_{0} & \cdots & z_{2} \end{array}\\ \sigma_{measure}=\left(\begin{array}{ccc} \epsilon (t) & \cdots & 0\\ \; & \ddots & \;\\ 0 & \cdots & \epsilon (t) \end{array}\right)\;\begin{array}{c} x_{0}\\ \vdots \\ z_{2} \end{array} \end{array}$$

The error propagation was calculated by applying the co-variance matrix to a numerically estimated Jacobian matrix (Equation 2) so σ_*bee*_ = *Jσ*_*measure*_. The Jacobian was numerically evaluated because of the large number of functions on which the expression of YPR of the head depends, this one derived from the three head markers' positions *xyz*_0_, *xyz*_1_, *xyz*_2_ (Equation 3). The propagated error does not only contain values on the diagonal, because the orientation of the bee is derived from the three markers.

(2) $$\begin{array}{l} \;J=\left(\begin{array}{ccccc} \frac{\partial f_{x}}{\partial x_{0}} & \frac{\partial f_{x}}{\partial y_{0}} & \frac{\partial f_{x}}{\partial z_{0}} & \cdots & \frac{\partial f_{x}}{\partial z_{2}}\\ \vdots \\ \frac{\partial f_{roll}}{\partial x_{0}} & \frac{\partial f_{roll}}{\partial y_{0}} & \frac{\partial f_{roll}}{\partial z_{0}} & \cdots & \frac{\partial f_{roll}}{\partial z_{2}} \end{array}\right)\\ \;\frac{\partial f_{x}}{\partial x_{0}}=\frac{f\left(x_{0}+h,y_{0},\cdots \;,z_{2}\right)-f\left(x_{0}-h,y_{0},\cdots \;,z_{2}\right)}{2h}\\ \text{where:}\\ \;h=10^{-6} \end{array}$$

(3) $$\begin{array}{l} \left(\begin{array}{c} {\text{x}}_{\text{bee}}\\ {\text{y}}_{\text{bee}}\\ {\text{z}}_{\text{bee}}\\ {\text{yaw}}_{\text{bee}}\\ {\text{pitch}}_{\text{bee}}\\ {\text{roll}}_{\text{bee}} \end{array}\right)=f\left(x_{m0},y_{m0},z_{m0},....x_{m2},y_{m2},z_{m2}\right)\\ \left(\begin{array}{c} {\text{x}}_{\text{bee}}\\ {\text{y}}_{\text{bee}}\\ {\text{z}}_{\text{bee}}\\ {\text{yaw}}_{\text{bee}}\\ {\text{pitch}}_{\text{bee}}\\ {\text{roll}}_{\text{bee}} \end{array}\right)=\left(\begin{array}{c} f_{x}\left(x_{0},...,z_{2}\right)\\ f_{y}\left(x_{0},...,z_{2}\right)\\ f_{z}\left(x_{0},...,z_{2}\right)\\ f_{yaw}\left(x_{0},...,z_{2}\right)\\ f_{pitch}\left(x_{0},...,z_{2}\right)\\ f_{roll}\left(x_{0},...,z_{2}\right) \end{array}\right) \end{array}$$

If rotations are observed around the z-axis during the intersaccadic intervals, it could be a result of our tracking error. Therefore, we estimated the significance of our measured yaw, by calculating its z-score. The z-score indicates the deviation of a value from the mean of a distribution. Here, we want to assess whether a measured orientation significantly differs from the average orientation during an intersaccade, or, in other words, whether our measure is significantly different from the hypothesis that the bee kept its head in the same orientation during the intersaccade. Thus, the null-hypothesis is that the observed deviation from the mean is due to an error. The z-score is the difference between the observed yaw and the circular mean of the yaw during the intersaccade, divided by the standard deviation of the propagated error. Thus, we can derive a measure of how likely the observed orientation at time *t* significantly differs from the mean. In this way, in (4), μ_*yaw*_ is the average yaw orientation during the intersaccade, hence representing an intersaccade with constant yaw (i.e., in the case of a stabilized head) and *error*, i.e., the propagated yaw-error at this time point. P-values are derived from the z-scores using Scipy.stats. A significant p-value means that the measure is significantly different from the error and therefore, is unlikely to be a consequence of it. All *p*-values are adjusted with the Bonferroni method to compensate for the effect of multiple comparisons.

(4) $$\begin{array}{l} Zscore\left(t\right)=\frac{\text{yaw(t)}-\mu }{std\left(error\left(t\right)\right)} \end{array}$$

**Figure 3:**
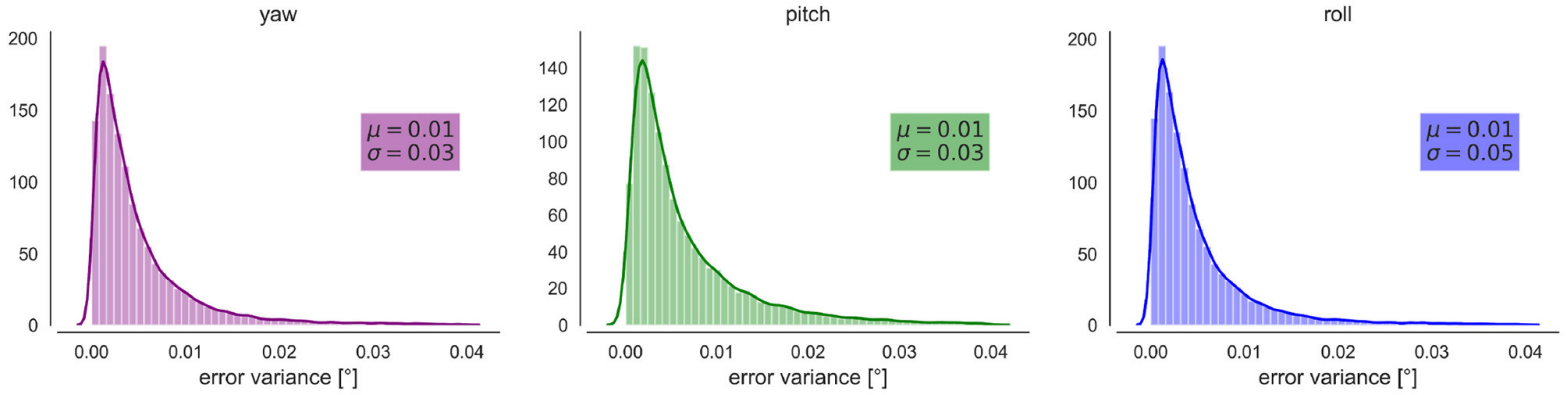
Propagated error on the YPR orientations. From Left to right Kernel Density Estimation and distribution of the errors (variance) for the yaw, pitch and roll orientations in degrees. μ indicates the mean and σ the standard deviation.

We observe a small error induced by our tracking method. In general, the error is below 0.1° for the three angles (Figure 3). 35% of the intersaccade have a variation statistically greater than variation due to measurement errors.

### 2.7. Optic-Flow Analysis

#### 2.7.1. Optic-Flow Calculation

The use of an active vision strategy requires the insect to control its head movement to shape its optic-flow (OF) in a specific manner. Thus, depending on which active vision strategy the insect is employing the OF will differ. Here, we solely focused on the OF resulting for the retinal displacement of the nest-hole's and the exit-hole's center. In a first step, we will explain how the OF was calculated; in a second step, we compare the experienced OF with the corresponding calculated OF induced by an ideal motion parallax or an ideal pivoting parallax, respectively.

For each projected point, the translational and rotational component of the OF, averaged along the intersaccadic interval, was determined following the equations given by Koenderink and van Doorn (1987). From the formula describing the displacement of a point (at the positions *Q*_*i*_) along time relative to the vantage point of an eye (Equation 5L1), the geometrical optical flow is the time derivative of the change in the direction of this point (see Equation 5L2). To ease computation Equation (5L2) is a simplification, since a dimensionless combination is formed from the speed and nearness (i.e; the product between speed and real nearness). Thus, nearness can be expressed as the “reduced nearness” (or also known as “time-to-contact”). In this expression, speed no longer plays a role, which makes it possible to solve the equation. The geometric OF can be found by reducing the number of unknown components (e.g., the three unknown rotational components, for detail see Koenderink and van Doorn, 1987). In Equation (2), *t* is a vector representing the direction of the translation, *d*_*i*_ is the viewing direction of the point, and μ_*i*_ is the reduced nearness. Finally 5 is written, such as in Equation (5L3), where *A*_*i*_ represents the “apparent rotation” due to a translation. Thus, Equation (5L3) separates the translational (*A*_*i*_) and rotational component (*R*_*i*_) of the flow field. This equation is expressed in a Cartesian coordinate system where the $\vec{OF}$ is a vector with the components *OF*_*x*_, *OF*_*y*_, and *OF*_*z*_ along *x*, *y*, and *z*-axis, respectively. $\vec{OF}$ can be converted to a spherical coordinate system to match the OF experienced by a spherical eye, following the method described in Bertrand et al. (2015). In our analysis, we focused on the representation of space, and thus on the distance to the nest entrance or the exit-hole, as derived from OF. These distances are linked to the amplitude of the $\vec{OF}$. Since the amplitude of a vector is independent of the coordinate system in which the vector is expressed, the chosen coordinate system, for example Cartesian or spherical, does not affect the results.

(5) $$\begin{array}{ll} \Delta {\text{Q}}_{\text{i}}=-\left(T+R\times Q_{i}\right)\delta \text{t} & \;\text{L1}\\ \;\vec{OF}=\mu_{i}\left(t-\left(t\times d_{i}\right)d_{i}\right)-R\times d_{i} & \;\text{L2}\\ \;\vec{OF}=-\left(A_{i}+R\right)\times d_{i} & \;\text{L3} \end{array}$$

#### 2.7.2. Assessment of the Signal-to-Noise-Ratio for Two Active Vision Strategies

**Figure 4:**
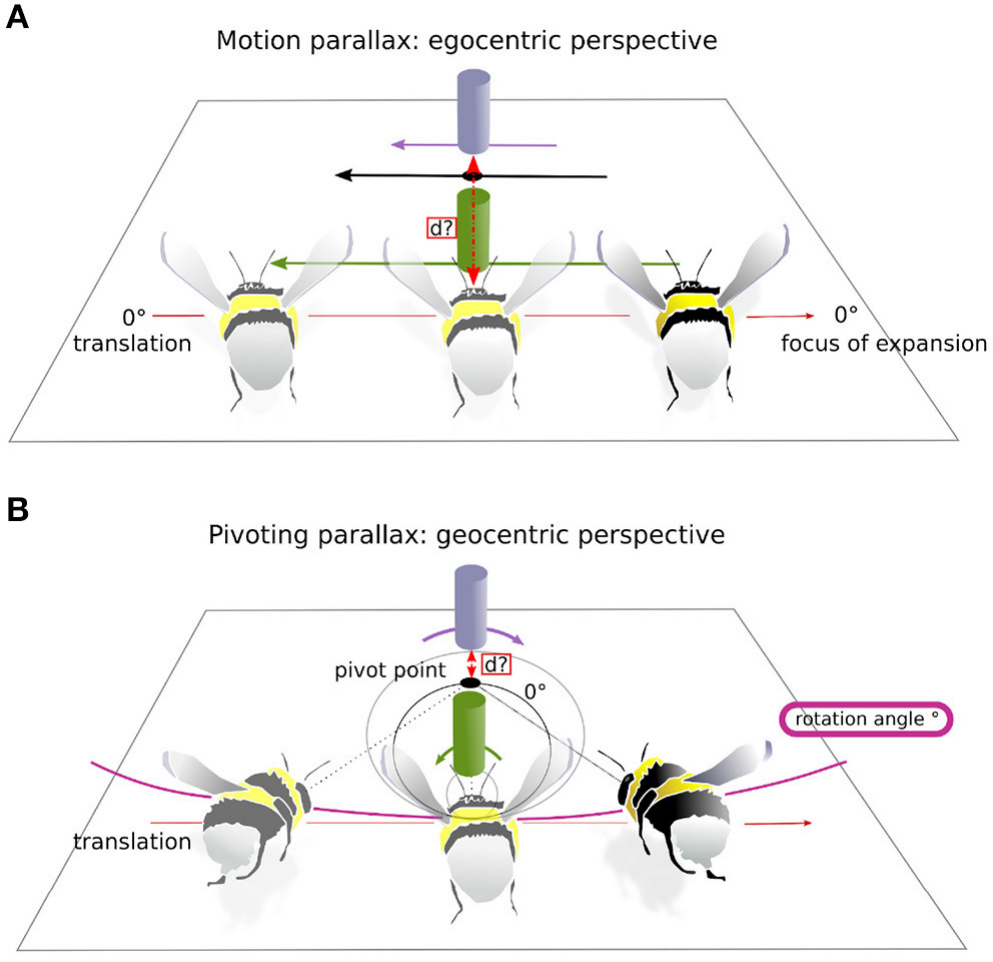
Two active vision strategies. This is a schematic of the two active vision strategies and their impact on the retinal displacement of visual landmarks on the retina. For illustrative purposes the head orientation is considered on the drawing aligned with the body axis. **(A)** Motion-parallax. As a consequence of translation the bumblebee gains distance information about the landmarks relative to its own current position. Here the purple landmark moves slower on the bumblebee's eye, shorter retinal displacement (purple arrow) than the green landmark, longer retinal displacement (green arrow). Thus, the purple object is more distant to the bumblebee. **(B)** Pivoting-parallax. The bumblebee pivots around a point, the pivot point, by a certain rotation angle while translating. By doing so the bumblebee gains distance information relative to the pivot point. Here, the purple landmark moves in the opposite direction on the retinae to the green landmark (see corresponding arrows of retinal displacement), because the latter is placed in between the pivot point and the bumblebee. The black circle represents the zero-horopter (as named in Zeil, 1993a), which separates areas of image motion with opposite sign: inside the horopter, the green landmark follows the rotation of the bumblebee and outside, the purple landmark moves in the opposite direction. Equation for the horopters is given in Zeil (1993a).

We wanted to investigate to what extent distance information is contained in the optic flow during intersaccadic intervals. Depending on the active vision strategy used, motion-parallax vs. pivoting-parallax, the resulting OF, and thus, the available distance information differs considerably (Figure 4). Motion parallax requires the head to be stabilized along its YPR axis. Thus, only translational movements of the head affect the OF. As a consequence, the nearest objects in the environment move faster on the retina, and the more distant ones slower (Figure 4A). Therefore, from the objects' apparent movement, distance information can be derived from an egocentric perspective. In contrast, pivoting parallax is characterized by the fixation of a fixed point in space, the pivoting point, and a uni-directional rotation of the head in the x-y plane around this point. As a consequence, distance can be estimated relative to the pivoting point, and thus, in a allocentric reference system (for details see Zeil, 1993a; Collett and Zeil, 1996, Figure 4B, and Supplementary Material). We computed for each intersaccadic interval the ideal flight trajectories and time-dependent head orientation that would be performed if bees were following one or the other active vision strategy. The resulting OF experienced from the two ideal trajectories is then compared to the OF derived from the recorded head movements. This comparison is formally expressed as a signal-to-noise-ratio (SNR) to distinguish between the two motion strategies. In the following the calculations of the two different ideal trajectories and of the SNR measures are described.

##### *2.7.2.1.* Ideal Motion-Parallax

We first calculated our signal, which is the OF obtained from a perfect execution of the motion parallax strategy. We simulated for a given intersaccade the ideal motion-parallax trajectory based on the measured x, y, and z coordinates of the bumblebee's head, but with its yaw, pitch and roll orientation assumed to be kept constant at the corresponding average values determined for the respective intersaccade. From this simulated trajectory, we determined the OF using the Equation (5L2). We calculated the OF induced by the nest-hole's and by the exit-hole's retinal projection because these are the only prominent behaviorally relevant and visually distinct locations in the flight arena.

##### *2.7.2.2.* Ideal pivoting-parallax

Assuming an ideal pivoting-parallax, we simulated for each intersaccade an ideal trajectory based on a uni-directional constant-velocity rotation of the head's yaw orientation around an unspecified pivoting point. This drift was obtained for each intersaccade by fitting a linear regression to the measured time-dependent yaw orientation. In this way, when an intersaccade is close to a null-drift (i.e., a null yaw rotational speed), the simulated trajectory will be similar to the one of a motion parallax strategy. A linear-fit may be worse than a null-drift in the data (for example, when an R-squared is negative). Therefore, it is possible to obtain a better SNR with a null-drift for some intersaccades.

##### *2.7.2.3.* Signal to noise ratio (SNR)

The signal to noise ratio indicates in the present context how well the perceived optic-flow by the bee correspond to the ideal optic-flow either due to motion-parallax or pivoting-parallax during an intersaccade. The signal is the amplitude of the average OF experienced during an ideal intersaccadic motion-parallax or pivoting-parallax, respectively. The noise is the absolute difference between the signal and the amplitude of the average OF experienced by the bee during the intersaccade. For both active gaze strategies and both points of interest (nest-entrance and exit-hole), we calculated the signal-to-noise ratio as in Equation (6). A log_10_ SNR smaller than 0 indicates the noise to be larger than the signal. We calculate the ratio between the SNR assuming a pivoting-parallax and motion parallax. Intersaccades with a ratio >1 were considered as pivoting-parallax.

(6) $$\begin{array}{l} \;SNR\left(i\right)=\frac{signal}{noise}=\frac{signal}{\left|signal-measure\right|}\\ SNR\left(i\right)=\frac{|\sum_{i}{\vec{OF}}_{ideal-motion\left(t\right)}{|}_{2}}{||\sum_{i}{\vec{OF}}_{ideal-motion\left(t\right)}{|}_{2}-|\sum_{i}{\vec{OF}}_{measure\left(t\right)}{|}_{2}{|}_{1}} \end{array}$$

where $\vec{OF}$ is the geometrical optic flow of the nest or exit-hole of the foraging chamber at time *t* of either the signal (pivoting parallax or motion parallax) or the measure during an intersaccade *i*. |...|_2_ and |...|_1_ indicate the Euclidian norm and absolute value, respectively.

#### 2.7.3. Determining Pivoting Points

**Figure 5:**
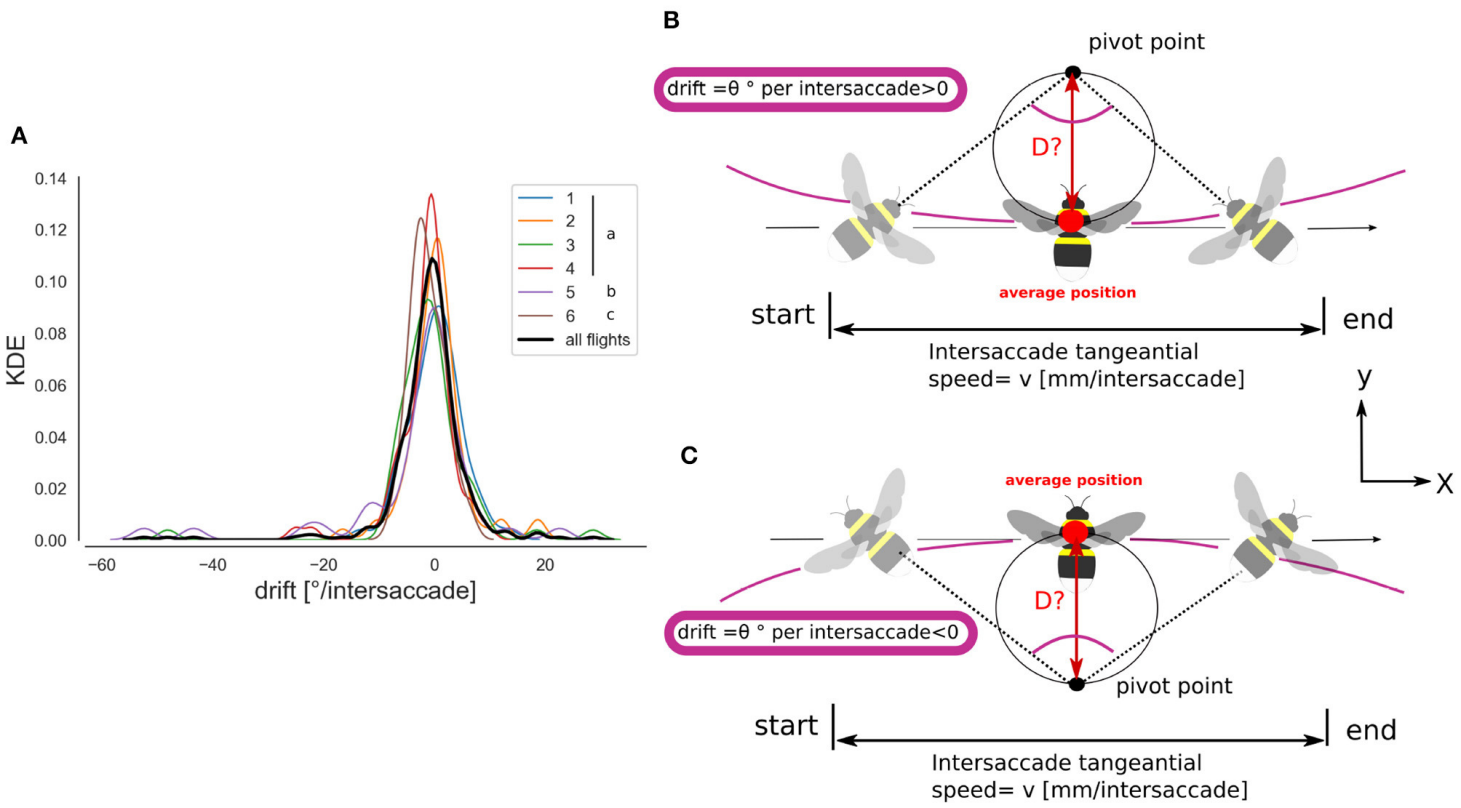
Intersaccade yaw drift and pivoting points location. **(A)** Kernel density estimation of the yaw drift during intersaccades expressed in degrees per intersaccade. KDE for all flights (thick black line) and for each flight (colored lines, see legend). **(B)** Schematic illustration of the method for estimating the pivot point location. With a positive rotational speed, or drift angle, the pivot point lies in the heading direction of the bee. Note that a pivoting-parallax can be due to head rotation only, therefore head-thorax are not necessarily aligned during a pivoting-parallax. **(C)** Negative drift angle, the pivot point lies behind the heading direction.

For intersaccades where the SNR was larger for the pivoting than for the motion parallax strategy, we estimated in two dimensions the location of the pivoting points. We did not account for the altitude to facilitate the computation as we found only relatively small variations along this spatial dimension. During pure motion-parallax characterized by a rotational drift of 0° per intersaccade, there is no pivoting point as the heading directions at the start and end of the intersaccade are parallel to each other and so cross at infinity. With a rotational drift different from 0° per intersaccade it is possible to calculate the pivoting point coordinates for an intersaccade of drift θ and tangential speed *v* following Equation (7). In this way, the pivoting point is in the heading direction of the bee if θ>0, and behind the bee (tail direction) if θ <0 (Figures 5A,B).

(7) $$\begin{array}{l} {\vec{x}}_{\text{pivot}}\left(i\right)=D\left(i\right)\left(\begin{array}{c} \cos\tilde{\alpha }\left(i\right)\\ \sin\tilde{\alpha }\left(i\right) \end{array}\right)+{\vec{\tilde{x}}}_{\text{bee}}\left(i\right)\\ \;D\left(i\right)=\frac{v}{2\tan\left(\theta \left(i\right)/2\right)} \end{array}$$

where ${\vec{x}}_{\text{pivot}}\left(i\right)$ is the position of the pivot-point at the i-th intersaccade, *D* is the distance between the bee and the pivot-point, *v* is the tangential speed during the intersaccade, θ is the drift during the intersaccade. The tilde represents the average during the intersaccade. α and ${\vec{x}}_{\text{bee}}$ are the yaw angle and position of the bee, respectively.

## 3. Results

### 3.1. Description of Head and Thorax Movements

#### 3.1.1. Yaw Saccadic Structure

**Figure 6:**
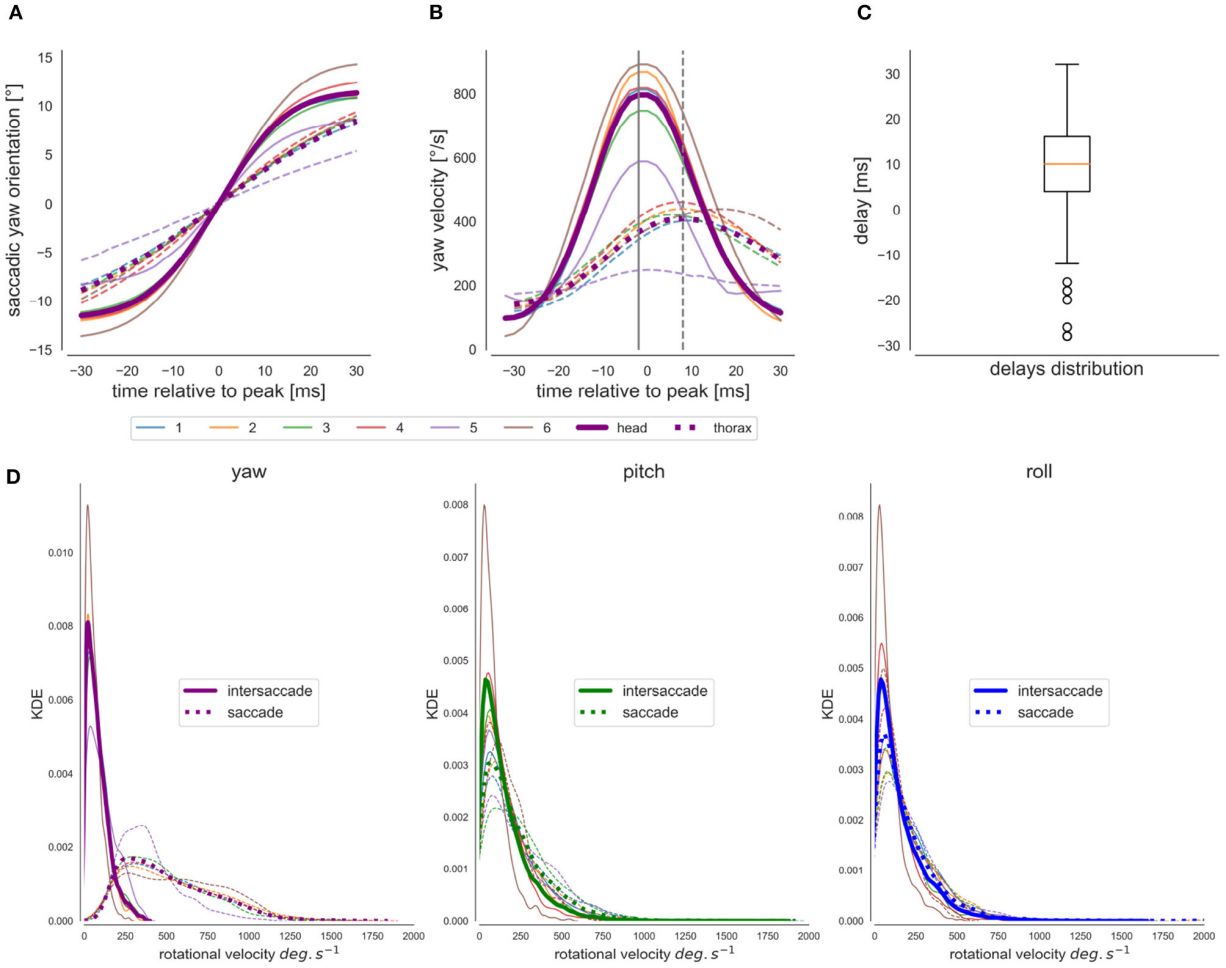
Analysis of head and thorax rotations. **(A)** Yaw orientation during saccades: head's yaw average purple line, thorax' s yaw-average dotted line, the different flights are individually colored [blue, orange green, and red (same bee “a”), purple and brown (bees “b” and “c”)]. **(B)** Average Yaw velocity during saccades. **(C)** Boxplot of the distribution of the thorax's velocity peak delay with the head during saccades, i.e., a negative value indicates a negative delay. **(D)** From left to right, distribution of yaw, pitch, and roll angular velocities for the head (*w*_*z*_, *w*_*y*_, *w*_*x*_, respectively) during saccades (dotted line) and intersaccades (continuous line).

The saccadic flight and gaze structure of learning flights of bumblebees is immediately visible when scrutinizing the time-dependant head and thorax yaw orientation. We can observe a distinct saccadic structure of the head's yaw rotations and a smoother one for the changes in the thorax's yaw orientation (Figures 2D,E). In the shown example, the saccadic structure of thorax movements fits the saccadic structure of the head movements temporally, but with apparently slower saccades and less stabilized yaw orientation during the intersaccadic intervals (Figure 2F) Furthermore, for a time window centered at the head saccades' velocity peak (time = 0 ms) (Figures 6A,B), we can see that the thorax initiates the saccadic yaw turns, and this for all flights. Interestingly, despite the thorax initiating the saccade, it reaches its velocity peaks, on average, 10 ms later than the head because the thorax turns more slowly (Figure 6C). The thorax, in general, performs a longer and slower saccade that is delayed to the head saccade's velocity peak. This observation is consistent with the concept of an active vision strategy where the thorax would initiate the saccade with the head following, confining head rotations to a minimal time interval. Interestingly, the bee performing flight nb° 5 shows an average yaw saccade of much smaller amplitude than the other bees (Figure 6D, thin purple line), suggesting inter-individual differences in the saccadic structure. Overall the head and the thorax are not always aligned during the flight as we can see from a closer look at one flight section with head and thorax orientation represented (Figures 2F,G).

Using a saccadic gaze strategy is generally associated with the assumption that the head direction is stabilized during the intersaccade. In the shown example, the yaw orientation during the intersaccadic intervals appears largely stabilized. During the intersaccade, the distribution of yaw velocities mostly lies between 0 and 400°· s^−1^, while yaw velocity distribution during saccadic intervals spreads between 0 and 2,000 °· s^−1^ (Figure 4D). We analyzed if the residual intersaccadic rotations are associated with low-frequency unidirectional turns, later called rotational drift. The distribution of angular amplitudes of the yaw drift angles during intersaccades can be well-approximated by a normal distribution (Agostino and Pearson: *p* <0.001) almost centered at zero (μ = −0.9°) with a standard deviation of 7.04° per intersaccade (Figure 5A). In Figure 2F, we show examples where the head orientation is performing strong rotational drift, either a positive or a negative one. This representative example reveals no clear relation between the learning flight structure and the occurrence of intersaccades with a strong rotational drift.

#### 3.1.2. Roll and Pitch Rotations

For the sample time course shown in Figure 2, but also for all other recorded flights (Supplementary Figure 1), the roll and pitch orientations vary around a constant value for both head and thorax. Overall during the saccades and similarly during intersaccades, the distribution of rectified roll and pitch velocities mostly lie between 0 and 250°· s^−1^ (Figure 6D).

### 3.2. SNR of the of at the Nest and Exit-Hole for an Assumed Pivoting or Motion-Parallax

Given the above described residual intersaccadic head rotations, the head may be actively rotated or stabilized to perform a pivoting or motion-parallax, respectively. Our results reveal unidirectional yaw rotations during many intersaccadic intervals hinting at an active gaze control to pivot around a point in space during the intersaccade (Figure 4B). We determined the SNR of the OF for an assumed motion parallax as well as for an assumed pivoting parallax for each intersaccade. We did this for two behaviorally relevant points in the arena, the exit-hole and the nest-hole.

**Figure 7:**
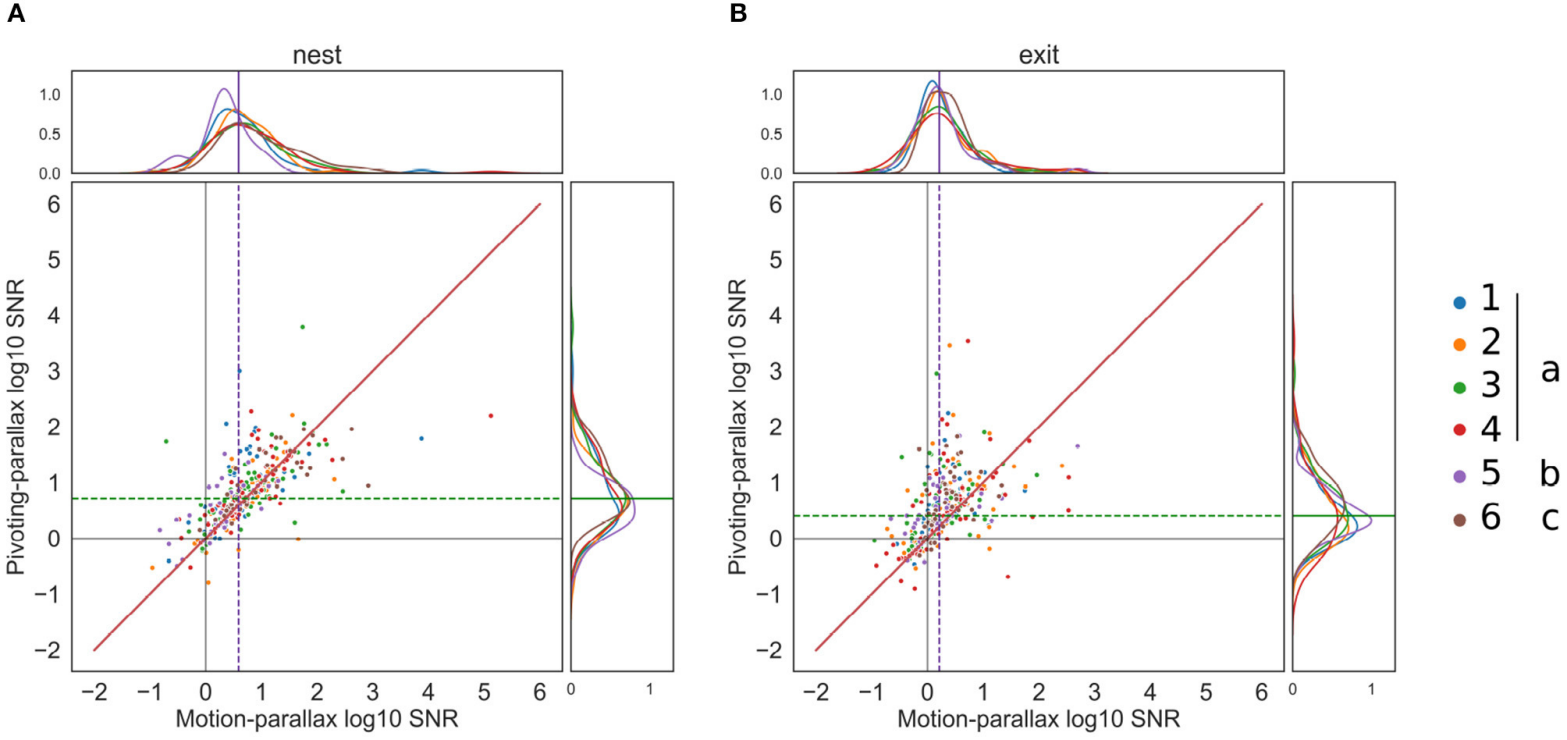
Pairwise comparison of the SNR for each intersaccadic interval. **(A)** Pairwise comparison of the SNR for the nest-hole retinal projection, for each flight (*n* = 6) color coded and with the different modifiers (none, roll constant, roll, and pitch constant). The motion-parallax SNR is on the x-axis and the pivoting-parallax SNR is on the y-axis; the bisection line is represented in red. The median log10 SNR for the pivoting and motion parallax are displayed with a green and purple dotted line, respectively. **(B)** same for the exit-hole SNR.

The pairwise comparison of the SNR obtained for both gaze strategies during each intersaccade for the nest-hole apparent motion, revealed a considerable variability. However, for some intersaccades, a larger SNR was obtained for a pivoting strategy (indicated by the points above the bisection line in Figure 7A). Overall the median SNR for the pivoting strategy is 5.26 and 3.9 for the motion parallax. This observation holds for all bees and flights, suggesting no idiosyncratic nor experience differences. For 57% of intersaccades, the SNR of the nest-hole's OF is larger for an assumed pivoting-parallax than for assumed motion-parallax.

The difference between the SNRs corresponding to the two gaze strategies is larger for the OF at the exit-hole to the feeding chamber (median pivoting's *SNR* = 2.53, median motion's *SNR* = 1.63). On the pairwise comparison (Figure 7B) some points considerably diverge toward pivoting and some toward motion-parallax, indicating that the SNR during some intersaccades is much better when considering a pivoting strategy while for others it is better when considering motion-parallax. The exit-hole's OF is closer to a pivoting strategy for 67% of the intersaccades. This finding raises the question where in space the pivoting point is located for these intersaccades.

### 3.3. Location of the Pivoting Points

Given that the nest-hole is kept in a broad frontal area of the visual field during most time of the learning flights, it is plausible to assume that the nest-hole is of particular functional significance for the bee. Therefore, it has been suggested that the pivoting points resulting from intersaccades characterized by an overall unidirectional rotation should be in the nest area (Riabinina et al., 2014). This would allow the bumblebee to derive a nest-area centered spatial representation of the nest surroundings. Our method, however, was not based on the assumption that the pivot-points are at the nest location.

**Figure 8:**
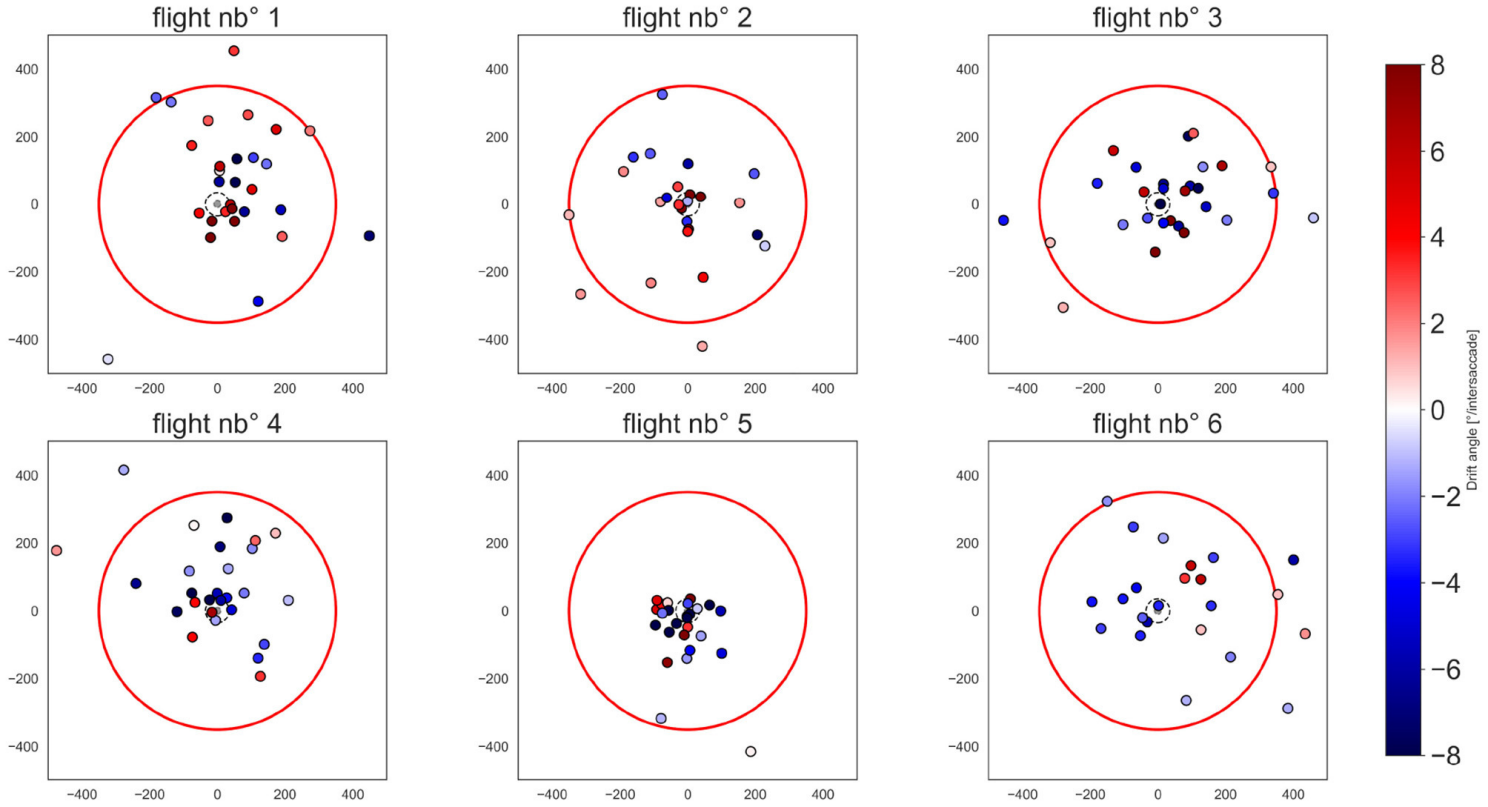
Pivoting points in the flight arena. Each subplot corresponds to one flight. Pivoting points are color-coded by a diverging color map depending on the drift angle of the corresponding intersaccade, i.e., a drift below 0, from white to blue; drift above 0 from white to red. The color map coloring is bounded to 8° for illustrative purposes. The arena walls are shown by the red circle. the nest-hole and platform are represented by the gray dot in the middle of the arena. The exit-hole is located at *x* = 0 and *y* = −350 mm.

Therefore, we determined the location of the pivoting points for each intersaccade (Figure 8). The distribution of the pivoting points in space is quite spread, though many of them cluster around the nest-hole, with 31.34% of them being within a radius of 10 cm from it. However, 30.34% of pivoting points lie outside the arena and 55.22% of pivoting points correspond to a negative drift and are, thus, located opposite to the heading direction of the bee (Figure 5C, blue points).

For the flights 1, 2, 3, and 4 belonging to the same individual and chronologically numbered, there are no apparent changes in the location of pivot points due to experience (Figure 8). Otherwise, it seems that there is an individual difference as flight 5 differs largely from the others. Flight nb° 5 has well-clustered pivot points around the nest-hole corresponding to intersaccades with relatively strong drifts.

## 4. Discussion

For navigating insects the problem of spatial representation of the environment and the corresponding reference frame, either egocentric or allocentric (which include nest-centric and geocentric), is a largely debated issue. Some researchers consider insects to be able to build geocentric cognitive maps, informing the insect about the relation between different objects in the environment (geocentric, Cheeseman et al., 2014), while for others the insect builds mainly egocentered representations of its environment (Cheung, 2014). Among the strategies used for homing, path-integration (PI) illustrates well the possible ambiguity of the different spatial representations (Heinze et al., 2018). PI is based on the integration of rotations about an external compass (an allocentric cue) and the speed of the animal obtained through egocentric information (idiothetic information) (Wehner et al., 1996; Heinze et al., 2018). In this way, the resulting home-vector, the direct line between the insect and its nest, can be considered to be egocentric and implicitly geocentric (in this case nest-centric) (Heinze et al., 2018; Webb, 2019).

In this study, we analyzed in which potential reference frame (egocentric or nest-centric) the depth structure of the nest surroundings is perceived during the bumblebees' learning flights. Therefore, we analyzed the head movements of bumblebees during learning flights around the three axes of rotation and investigated closely the period of head relative stabilization. We perform the recordings in a rather barren habitat where only two visual landmarks were present the exit-hole and the nest-hole.

### 4.1. Bumblebee's Head Stabilization

The saccadic flight and gaze strategy of insects is thought to be characterized by largely stabilized pitch and roll angles of the head, with residual head rotations being relatively small. Larger pitch and roll variation induce larger rotational component in optic-flow. Such component are independent of the spatial layout and may impair the depth perception. This kind of head stabilization has been observed in semi-free flying blowflies (Van Hateren and Schilstra, 1999) where during intersaccades angular rotations along YPR are largely high-frequency and smaller than 100° per seconds, which is much less than the corresponding rotational velocities we found for bumblebees (Figure 6D). This difference could be explained by Dipterans possessing halteres, organs acting like a gyroscope, which could contribute to compensatory head stabilization during intersaccades thanks to the small delay times of the mechano-sensory system (Hengstenberg, 1993). However, also the smaller body-size of the blowfly and the corresponding smaller inertia in comparison to bumblebees may play a role in this context (Boeddeker et al., 2015). Despite the relatively large angular roll velocities of the bumblebee head even during intersaccades, the head roll orientation in world coordinates was still stabilized to a large extent in comparison to the thorax (Figure 2). In this regard, our data are similar to observations on honeybees (Boeddeker and Hemmi, 2010) and wasps (Viollet and Zeil, 2013), although, in these investigations, the recorded flights were much simpler than the complex loop characteristic of learning flights recorded in this study. Moreover, since our analysis was done in an indoor set-up, whereas the wasps were recorded outdoor (Viollet and Zeil, 2013), the similar results obtained for intersaccadic head roll stabilization are most likely not the consequence of visual cues, such as a light gradient or polarized light which could be processed by the ocelli (Viollet and Zeil, 2013; Hardcastle and Krapp, 2016). All-together, our findings do not much differ from other hymenopteran species, and we can thus assume that even during complex learning flights, the spatial information contained in the OF will not be more impaired during learning flights than during other less complex behaviors.

### 4.2. Yaw Rotations During Intersaccade

It has been proposed that bumblebees could collect distance information about the visual surroundings of the nest location in the course of learning flights during the intersaccadic intervals by controlling in a specific way their yaw rotations. Bumblebees were suggested to generate a unidirectional yaw rotation around the position of the nest acting as a pivoting point (Riabinina et al., 2014). Thus, distances extracted from the resulting OF would be about the nest-hole as a potential basis of a nest-centered representation of the depth of the environment.

We investigated the yaw rotations during intersaccadic intervals during learning flights to classify these as being either induced by a motion-parallax strategy (Figure 4A) or a pivoting strategy (Figure 4B), and thus, respectively associated to either an egocentric or nest-centric representation of space. Bumblebees often performed unidirectional head yaw rotations during intersaccadic intervals (Figure 5A) and half of the intersaccadic intervals could be classified as a result of a pivoting strategy (Figure 7). However, these intersaccades often correspond to pivot points largely spread in the environment and even behind the bumblebees (Figures 5C, 8). Thus, the pivoting point is not in the nest region in most cases. This finding implies that at least for these flight sections, the distance information available in the OF pattern cannot be easily related to the location of the nest-hole. Therefore, questions arise about the potential functional consequences of such rotations for navigation during learning flights and for future homing trips.

### 4.3. Implications of the Reference Frame for Homing

Intuitively, information about the spatial structure of the environment that is memorized during the bees' learning flights should be related to the nest, which would then be the reference point. Depending on the active vision strategy used, the bee may need to link the collected spatial information of the environment to geocentric coordinates. This would be computationally the easiest if the bees perform a pivoting parallax around the nest-hole. Then the distance of objects in its environment were immediately represented in the retinal OF pattern in a nest-centered fashion. In contrast, if the bee would employ a motion-parallax strategy close to the nest-hole, it could immediately derive from the OF only egocentric distance information. Nest-hole centered spatial information would have to be computed requiring information about the bee's own position in a geocentric reference frame. The situation would be also computationally demanding for the pivoting parallax strategy if the location of the pivot point is not close to the nest location. Then information about its relation to the nest-hole location is required. However, neither information about the bee's own position in space nor that about another point, such as the pivot point, can be acquired easily, in particular, because of the complex choreography of learning flights (Cheung, 2014). Moreover, it would require that bumblebees can visually follow their nest and deduce their distance and orientation to it (Schulte et al., 2019). These conclusions may question the ability of bumblebees to easily use distance information contained in the OF for navigational purposes like local homing.

Yet, several modeling analyses showed that to enable successful homing, there is no need to know the exact location in space where the bee memorizes environmental information, such as a panoramic-snapshot (Dewar et al., 2014; Doussot et al., 2020a). These models solely require the memorized panoramic-snapshots to be oriented toward the nest-hole. This orientation could be obtained by visually tracking the entrance to the nest, or by using external compass cue (Fleischmann et al., 2018; Schulte et al., 2019). These conclusions make it quite unlikely that the bumblebee determines any map centered at the nest location, which would be computationally costly for the animal. However, even when employing the kind of snapshot strategy mentioned above, the bee should stabilize its head along the roll and pitch axes while gathering these snapshots, which is not exactly what we observed. Rotations along the roll and pitch axes have been shown to impair route-following behavior based on a use of brightness snapshot [effects of roll (Raderschall et al., 2016), and pitch variations (Ardin et al., 2015)]. The use of panoramic snapshots should be combined with either visual processing invariant against roll and pitch rotations [e.g., by using spherical harmonics or Zernike moments (Stone et al., 2016, 2018)], a large number of snapshots taken at the same location to average out the noise introduced by uncontrolled head rotations (Ardin et al., 2015), or limiting learning to moments of head stabilization. Indeed, the roll and pitch orientation of the head could be inferred from the horizon and light gradients possibly perceived by the ocelli [in locust (Taylor, 1981)].

### 4.4. Tracking of the Head Orientation During Free-Flight and Method Limitations

Our conclusions must be brought into line with the limitations of our method: our sample size is relatively small, and reconstruction errors cannot be fully avoided (Figure 3). Indeed, the placement of markers is challenging due to the small head of bumblebees covered with thick hair, which limits the number of individuals keeping the markers when placed back in the hive. Besides, the positioning of markers needs to be precisely controlled. When the placement differs between individuals, the geometrical relation between the markers and the head will impact the obtained YPR orientations, which would makes a comparison between animals difficult. This might be a reason that the saccades of individual “b” (=flight nb° 5) appear to be smaller than those of the other tested animals.

As a consequence of these technical constraints, we could analyse the head orientation only within a small volume of space, and thus, only for the initial section of the learning flights, while these flights are known to be naturally much longer (Degen et al., 2018). Overall, we largely reduced the reconstruction error to a minimum by using footage obtained at a high spatial-temporal resolution from multiple cameras and by appropriate filtering. In any case, our main conclusions are consistent across all flights and individuals: the head was only imperfectly stabilized along the YPR axes during intersaccades with the potential functional consequences for the reference frame for OF-based spatial information. Moreover, the head performed much faster rotations than the thorax extending the duration of the intersaccadic intervals. In consequence, using the thorax orientation as a proxy for head orientation would only deliver a poor estimate of the OF pattern experienced by the insect. Due to the methodological difficulties imposed by head tracking, it would be of interest to determine an algorithm, which from the less challenging measurements of thorax YPR's orientation could provide a good prediction about the head orientation (Kern et al., 2006; Odenthal et al., 2020).

Overall the observations made in our environment cannot be concluded to be representative of learning flights under other environmental conditions. Our environment offers little visual landmarks, while natural surroundings may be much more cluttered. It would therefore be interesting to analyse whether the active vision strategies, pivoting or motion parallax, are present to a different extent when the visual surroundings are more complex. In addition to visual information, natural environments provide other sensory cues that could be relevant while pinpointing and learning the nest-hole (Buehlmann et al., 2020; Vega Vermehren et al., 2020). One possibility might be an odor plume emanating from the nest-hole, as described in ants using an odor vector in combination to path-integration (Buehlmann et al., 2012). Therefore, the choreography of learning and homing flights could be influenced by such an odor plume in a similar manner as male moths zigzagging toward a female emitting pheromones (Murlis et al., 1992; Cardé and Willis, 2008).

## 5. Conclusion

In summary, despite the above discussed limitations, we were able to monitor head movements of bumblebees at unprecedented spatial resolution. However, even on this basis, we could not obtain evidence that bumblebees might gather views in a nest-centered reference frame. Moreover, we do not have a solid basis to conclude that the residual head rotations during intersaccades are actively controlled according to a pivoting strategy allowing for a geocentric representation of space, or that the intersaccadic rotations are the result of a poor execution of a motion-parallax strategy. It may well be possible that both spatial representations might co-exist and that bumblebees are able to use both kind of strategies depending on the behavioral context.

This study highlights that head movements of bees in flight, even if small, can radically change the perception of space. It is therefore necessary to study how active vision strategies might be affected by tethering the thorax and/or the head while walking stationary on trackballs or flying in flight simulators, coupled to virtual reality (VR) displays (Schultheiss et al., 2017; Kaushik and Olsson, 2020). For example, Drosophila head movements allow them to follow figures on a moving background, a behavior that is impaired when the head is held fixed (Fox and Frye, 2014). Based on our results, it seems necessary to reflect on how virtual reality scenarios should be used in relation to head movements and not only in relation to the body. The marking procedure proposed in our study might facilitate this technical feat. As an alternative, transfer experiments from setting allowing for free-behaving animals to VR (Buatois et al., 2018; Goulard et al., 2020) might enable to compensate for the potential loss of visual information induced by a restricted head and thorax in tethered animals.

## Data Availability Statement

The data and codes are available in the following repository: doi: 10.4110/unibi/2945856, https://pub.uni-bielefeld.de/record/2945856.

## Author Contributions

ME, CD, and OB conceptualized the project. ME applied for the funding of the project. CD designed the bumblebee experiments and wrote the manuscript. CD and OB built the set-up, conceived and supervised the experiments, conceived the methodology, and performed the formal analysis. All authors contributed to manuscript revision, read, and approved the submitted version.

## Conflict of Interest

The authors declare that the research was conducted in the absence of any commercial or financial relationships that could be construed as a potential conflict of interest.

## References

[B1] Ardin P. Mangan M. Wystrach A. Webb B. (2015). How variation in head pitch could affect image matching algorithms for ant navigation. J. Compar. Physiol. A Neuroethol. Sens. Neural Behav. Physiol. 201, 585–597. 10.1007/s00359-015-1005-8 25895895 PMC4439443

[B2] Avraamides M. N. Kelly J. W. (2008). Multiple systems of spatial memory and action. Cogn. Process. 9, 93–106. 10.1007/s10339-007-0188-5 17899235

[B3] Baddeley B. Philippides A. Graham P. De Ibarra N. H. Collett T. Husbands P. (2009). What can be learnt from analysing insect orientation flights using probabilistic Slam? Biol. Cybern. 101, 169–182. 10.1007/s00422-009-0327-4 19639335

[B4] Bertrand O. J. Lindemann J. P. Egelhaaf M. (2015). A bio-inspired collision avoidance model based on spatial information derived from motion detectors leads to common routes. PLoS Comput. Biol. 11:e1004339. 10.1371/journal.pcbi.1004339 26583771 PMC4652890

[B5] Boeddeker N. Dittmar L. Stürzl W. Egelhaaf M. (2010). The fine structure of honeybee head and body yaw movements in a homing task. Proc. R. Soc. B Biol. Sci. 277, 1899–1906. 10.1098/rspb.2009.2326 20147329 PMC2871881

[B6] Boeddeker N. Hemmi J. M. (2010). Visual gaze control during peering flight manoeuvres in honeybees. Proc. R. Soc. B Biol. Sci. 277, 1209–1217. 10.1098/rspb.2009.1928 20007175 PMC2842814

[B7] Boeddeker N. Mertes M. Dittmar L. Egelhaaf M. (2015). Bumblebee homing: the fine structure of head turning movements. PLoS ONE 10:e0135020. 10.1371/journal.pone.0135020 26352836 PMC4564262

[B8] Buatois A. Flumian C. Schultheiss P. Avarguès-Weber A. Giurfa M. (2018). Transfer of visual learning between a virtual and a real environment in honey bees: the role of active vision. Front. Behav. Neurosci. 12:139. 10.3389/fnbeh.2018.00139 30057530 PMC6053632

[B9] Buehlmann C. Hansson B. S. Knaden M. (2012). Path integration controls nest-plume following in desert ants. Curr. Biol. 22, 645–649. 10.1016/j.cub.2012.02.029 22405868

[B10] Buehlmann C. Mangan M. Graham P. (2020). Multimodal interactions in insect navigation. Anim. Cogn. 23, 1129–1141. 10.1007/s10071-020-01383-2 32323027 PMC7700066

[B11] Burgess N. (2006). Spatial memory: how egocentric and allocentric combine. Trends Cogn. Sci. 201, 585–597. 10.1016/j.tics.2006.10.005 17071127

[B12] Cardé R. T. Willis M. A. (2008). Navigational strategies used by insects to find distant, wind-borne sources of odor. J. Chem. Ecol. 34, 854–866. 10.1007/s10886-008-9484-5 18581182

[B13] Cheeseman J. F. Millar C. D. Greggers U. Lehmann K. Pawley M. D. M. Gallistel C. R.. (2014). Reply to cheung et al.: the cognitive map hypothesis remains the best interpretation of the data in honeybee navigation. Proc. Natl. Acad. Sci. U.S.A. 111:E4398. 10.1073/pnas.1415738111 25277971 PMC4210344

[B14] Cheung A. (2014). Animal path integration: a model of positional uncertainty along tortuous paths. J. Theor. Biol. 341, 17–33. 10.1016/j.jtbi.2013.09.031 24096099

[B15] Cheung A. Vickerstaff R. (2010). Finding the way with a noisy brain. PLoS Comput. Biol. 6:e1000992. 10.1371/journal.pcbi.1000992 21085678 PMC2978673

[B16] Cheung A. Zhang S. Stricker C. Srinivasan M. V. (2007). Animal navigation: the difficulty of moving in a straight line. Biol. Cybern. 97, 47–61. 10.1007/s00422-007-0158-0 17520273

[B17] Collett T. S. Zeil J. (1996). Flights of learning. Curr. Direct. Psychol. Sci. 5, 149–155. 10.1111/1467-8721.ep11512352

[B18] Collett T. S. Zeil J. (2018). Insect learning flights and walks. Curr. Biol. 28, R984–R988. 10.1016/j.cub.2018.04.050 30205076

[B19] Degen J. Hovestadt T. Storms M. Menzel R. (2018). Exploratory behavior of re-orienting foragers differs from other flight patterns of honeybees. PLoS ONE 13:e0202171. 10.1371/journal.pone.0202171 30157186 PMC6114720

[B20] Dewar A. D. Philippides A. Graham P. (2014). What is the relationship between visual environment and the form of ant learning-walks? An *in silico* investigation of insect navigation. Adapt. Behav. 22, 163–179. 10.1177/1059712313516132

[B21] Diebel J. (2006). Representing attitude: Euler angles, unit quaternions, and rotation vectors. Matrix 58, 15–16.

[B22] Dittmar L. Egelhaaf M. Stürzl W. Boeddeker N. (2011). The behavioral relevance of landmark texture for honeybee homing. Front. Behav. Neurosci. 5:20. 10.3389/fnbeh.2011.00020 21541258 PMC3083717

[B23] Dittmar L. Sturzl W. Baird E. Boeddeker N. Egelhaaf M. (2010). Goal seeking in honeybees: matching of optic flow snapshots? J. Exp. Biol. 213, 2913–2923. 10.1242/jeb.043737 20709919

[B24] Doussot C. Bertrand O. Egelhaaf M. (2020a). Visually guided homing of bumblebees in ambiguous situations: a behavioural and modelling study. PLoS Comput. Biol. 16:e1008272. 10.1371/journal.pcbi.1008272 33048938 PMC7553325

[B25] Doussot C. Odenthal L. Meyer S. Egelhaaf M. Bertrand O. J. (2020b). Head-Thorax Orientation of Bombus Terrestris During Learning Flights. Bielefeld: University of Bielefeld 10.4119/unibi/2946065

[B26] Eckmeier D. Geurten B. Kress D. Mertes M. Kern R. Egelhaaf M. (2008). Gaze strategy in the free flying zebra finch (*Taeniopygia guttata*). PLoS ONE 3:e3956 10.1371/journal.pone.0003956 19107185 PMC2600564

[B27] Egelhaaf M. Boeddeker N. Kern R. Kurtz R. Lindemann J. P. (2012). Spatial vision in insects is facilitated by shaping the dynamics of visual input through behavioral action. Front. Neural Circuits 6:108. 10.3389/fncir.2012.00108 23269913 PMC3526811

[B28] Fleischmann P. (2019). Starting foraging life: early calibration and daily use of the navigational system in (Cataglyphis) ants (Ph.D. thesis), Universität Wurzburg, Würzburg, Germany.

[B29] Fleischmann P. N. Grob R. Müller V. L. Wehner R. Rössler W. (2018). The geomagnetic field is a compass cue in cataglyphis ant navigation. Curr. Biol. 28, 1440–1444. 10.1016/j.cub.2018.03.043 29706513

[B30] Fox J. L. Frye M. A. (2014). Figure-ground discrimination behavior in Drosophila. II. Visual influences on head movement behavior. J. Exp. Biol. 217, 558–569. 10.1242/jeb.097220 24198264 PMC3922834

[B31] Fry S. N. Wehner R. (2002). Honey bees store landmarks in an egocentric frame of reference. J. Compar. Physiol. A Sens. Neural Behav. Physiol. 187, 1009–1016. 10.1007/s00359-001-0272-8 11913810

[B32] Geurten B. R. Kern R. Braun E. Egelhaaf M. (2010). A syntax of hoverfly flight prototypes. J. Exp. Biol. 213, 2461–2475. 10.1242/jeb.036079 20581276

[B33] Goulard R. Buehlmann C. Niven J. E. Graham P. Webb B. (2020). Transfer of orientation memories in untethered wood ants (*Formica rufa*) from walking in an arena to walking on a motion compensation treadmill. bioRxiv. 10.1101/2020.05.29.084905 PMC7774907 33443039

[B34] Hardcastle B. J. Krapp H. G. (2016). Evolution of biological image stabilization. Curr. Biol. 26, 1010–1021. 10.1016/j.cub.2016.08.059 27780044

[B35] Hedrick T. L. (2008). Software techniques for two- and three-dimensional kinematic measurements of biological and biomimetic systems. Bioinspir. Biomimet. 3:034001. 10.1088/1748-3182/3/3/034001 18591738

[B36] Heinze S. Narendra A. Cheung A. (2018). Principles of insect path integration. Curr. Biol. 28, 1043–1058. 10.1016/j.cub.2018.04.058 30205054 PMC6462409

[B37] Hengstenberg R. (1993). Multisensory control in insect oculomotor systems. Rev. Oculom. Res. 5, 285–288. 8420553

[B38] Hoinville T. Wehner R. (2018). Optimal multiguidance integration in insect navigation. Proc. Natl. Acad. Sci. U.S.A. 115, 2824–2829. 10.1073/pnas.1721668115 29483254 PMC5856556

[B39] Hugues I. Hase T. (2010). Measurements and Their Uncertainties: A Practical Guide to Modern Error Analysis. Oxford: Oxford University Press Inc.

[B40] Kaushik P. K. Olsson S. B. (2020). Using virtual worlds to understand insect navigation for bio-inspired systems. Curr. Opin. Insect Sci. 42, 97–104. 10.1016/j.cois.2020.09.010 33010476

[B41] Kern R. Boeddeker N. Dittmar L. Egelhaaf M. (2012). Blowfly flight characteristics are shaped by environmental features and controlled by optic flow information. J. Exp. Biol. 215, 2501–2514. 10.1242/jeb.061713 22723490

[B42] Kern R. van Hateren J. H. Egelhaaf M. (2006). Representation of behaviourally relevant information by blowfly motion-sensitive visual interneurons requires precise compensatory head movements. J. Exp. Biol. 209, 1251–1260. 10.1242/jeb.02127 16547297

[B43] Koenderink J. J. van Doorn A. J. (1987). Facts on optic flow. Biol. Cybern. 56, 247–254. 10.1007/BF00365219 3607100

[B44] Lobecke A. Kern R. Egelhaaf M. (2018). Taking a goal-centred dynamic snapshot as a possibility for local homing in initially naïve bumblebees. J. Exp. Biol. 221. 10.1242/jeb.168674 29150448

[B45] Mou W. McNamara T. P. Rump B. Xiao C. (2006). Roles of egocentric and allocentric spatial representations in locomotion and reorientation. J. Exp. Psychol. Learn. Mem. Cogn. 32, 1274–1290. 10.1037/0278-7393.32.6.1274 17087583

[B46] Mronz M. Lehmann F.-O. (2008). The free-flight response of Drosophila to motion of the visual environment. J. Exp. Biol. 211, 2026–2045. 10.1242/jeb.008268 18552291

[B47] Muller M. Wehner R. (1988). Path integration in desert ants, *Cataglyphis fortis*. Proc. Natl. Acad. Sci. U.S.A. 85, 5287–5290. 10.1073/pnas.85.14.5287 16593958 PMC281735

[B48] Murlis J. Elkinton J. S. Carde R. T. (1992). Odor plumes and how insects use them. Annu. Rev. Entomol. 37, 505–532. 10.1146/annurev.en.37.010192.002445

[B49] Nicholson D. J. Judd S. P. Cartwright B. A. Collett T. S. (1999). Learning walks and landmark guidance in wood ants (*Formica rufa*). J. Exp. Biol. 202, 1831–1838. 10359685 10.1242/jeb.202.13.1831

[B50] Odenthal L. Doussot C. Meyer S. Bertrand O. J. (2020). Analysing head-thorax relation during free-flights in bumblebees. Front. Behav. Neurosci. 10.3389/fnbeh.2020.610029 PMC7835495 33510626

[B51] Pete A. E. Kress D. Dimitrov M. A. Lentink D. (2015). The role of passive avian head stabilization in flapping flight. J. R. Soc. Interface 12:20150508. 10.1098/rsif.2015.0508 26311316 PMC4614461

[B52] Philippides A. de Ibarra N. H. Riabinina O. Collett T. S. (2013). Bumblebee calligraphy: the design and control of flight motifs in the learning and return flights of *Bombus terrestris*. J. Exp. Biol. 216, 1093–1104. 10.1242/jeb.081455 23447668

[B53] Raderschall C. A. Narendra A. Zeil J. (2016). Head roll stabilisation in the nocturnal bull ant *Myrmecia pyriformis*: implications for visual navigation. J. Exp. Biol. 219, 1449–1457. 10.1242/jeb.134049 26994172

[B54] Ravi S. Crall J. D. Fisher A. Combes S. A. (2013). Rolling with the flow: bumblebees flying in unsteady wakes. J. Exp. Biol. 216, 4299–4309. 10.1242/jeb.090845 24031057

[B55] Ravi S. Crall J. D. McNeilly L. Gagliardi S. F. Biewener A. A. Combes S. A. (2015). Hummingbird flight stability and control in freestream turbulent winds. J. Exp. Biol. 218, 1444–1452. 10.1242/jeb.114553 25767146

[B56] Riabinina O. de Ibarra N. H. Philippides A. Collett T. S. (2014). Head movements and the optic flow generated during the learning flights of bumblebees. J. Exp. Biol. 217, 2633–2642. 10.1242/jeb.102897 25079890

[B57] Robert T. Frasnelli E. de Ibarra N. H. Collett T. S. (2018). Variations on a theme: bumblebee learning flights from the nest and from flowers. J. Exp. Biol. 221:jeb172601. 10.1242/jeb.172601 29361597

[B58] Ros I. G. Biewener A. A. (2017). Pigeons (*C. livia*) follow their head during turning flight: head stabilization underlies the visual control of flight. Front. Neurosci. 11:655. 10.3389/fnins.2017.00655 29249929 PMC5717024

[B59] Schulte P. Zeil J. Stürzl W. (2019). An insect-inspired model for acquiring views for homing. Biol. Cybern. 113, 439–451. 10.1007/s00422-019-00800-1 31076867

[B60] Schultheiss P. Buatois A. Avarguès-Weber A. Giurfa M. (2017). Using virtual reality to study visual performances of honeybees. Curr. Opin. Insect Sci. 24, 43–50. 10.1016/j.cois.2017.08.003 29208222

[B61] Serres J. R. Ruffier F. (2017). Optic flow-based collision-free strategies: from insects to robots. Arthropod. Struct. Dev. 46, 703–717. 10.1016/j.asd.2017.06.003 28655645

[B62] Stone T. Differt D. Milford M. Webb B. (2016). Skyline-based localisation for aggressively manoeuvring robots using UV sensors and spherical harmonics, in Proceedings–IEEE International Conference on Robotics and Automation, (Stockholm) 5615–5622. 10.1109/ICRA.2016.7487780

[B63] Stone T. Mangan M. Wystrach A. Webb B. (2018). Rotation invariant visual processing for spatial memory in insects. Interface Focus 277, 1209–1217. 10.1098/rsfs.2018.0010 PMC6015815 29951190

[B64] Sun X. Yue S. Mangan M. (2020). A decentralised neural model explaining optimal integration of navigational strategies in insects. eLife 9:e54026. 10.7554/eLife.54026.sa2 32589143 PMC7365663

[B65] Taylor C. P. (1981). Contribution of compound eyes and ocelli to steering of locusts in flight: II. Timing changes in flight motor units. J. Exp. Biol. 93, 1–18.

[B66] Van Hateren J. H. Schilstra C. (1999). Blowfly flight and optic flow. II. Head movements during flight. J. Exp. Biol. 202, 1491–1500. 10229695 10.1242/jeb.202.11.1491

[B67] Vardy A. Möller R. (2005). Biologically plausible visual homing methods based on optical flow techniques. Connect. Sci. 17, 47–89. 10.1080/0954009050014095818804202

[B68] Vega Vermehren J. A. Buehlmann C. Fernandes A. S. D. Graham P. (2020). Multimodal influences on learning walks in desert ants (*Cataglyphis fortis*). J. Compar. Physiol. A Neuroethol. Sens. Neural Behav. Physiol. 206, 701–709. 10.1101/2020.04.17.046839 32537664 PMC7392947

[B69] Viollet S. Zeil J. (2013). Feed-forward and visual feedback control of head roll orientation in wasps (polistes humilis, vespidae, hymenoptera). J. Exp. Biol. 216, 1280–1291. 10.1242/jeb.074773 23239889

[B70] Webb B. (2019). The internal maps of insects. J. Exp. Biol. 222:jeb188094. 10.1242/jeb.188094 30728234

[B71] Wehner R. (2009). The architecture of the desert ant's navigational toolkit (Hymenoptera: Formicidae). Myrmecol. News 55, 101–114. 10.1002/j.2161-4296.2008.tb00421.x

[B72] Wehner R. Michel B. Antonsen P. (1996). Visual navigation in insects: coupling of egocentric and geocentric information. J. Exp. Biol. 199, 129–140. 9317483 10.1242/jeb.199.1.129

[B73] Woltring H. J. (1985). On optimal smoothing and derivative estimation from noisy displacement data in biomechanics. Hum. Mov. Sci. 4, 229–245. 10.1016/0167-9457(85)90004-1

[B74] Wystrach A. Mangan M. Webb B. (2015). Optimal cue integration in ants. Proc. R. Soc. B Biol. Sci. 282:20151484. 10.1098/rspb.2015.1484 26400741 PMC4614770

[B75] Zeil J. (1993a). Orientation flights of solitary wasps (Cerceris; Sphecidae; Hymenoptera): I. Description of flight. J. Compar. Physiol. A Sens. Neural Behav. Physiol. 172, 189–205. 10.1007/BF00189396

[B76] Zeil J. (1993b). Orientation flights of solitary wasps (Cerceris; Sphecidae; Hymenoptera): II. Similarities between orientation and return flights and the use of motion parallax. J. Compar. Physiol. A Sens. Neural Behav. Physiol. 172, 207–222. 10.1007/BF00189397

[B77] Zeil J. (2012). Visual homing: an insect perspective. Curr. Opin. Neurobiol. 22, 285–293. 10.1016/j.conb.2011.12.008 22221863

