# Supplementary material for: The Critical Role of Head Movements for Spatial Representation During Bumblebees Learning Flight
Source: Front Behav Neurosci. 2021 Jan 19;14:606590. doi: 10.3389/fnbeh.2020.606590 (PMC7852487; doi:10.3389/fnbeh.2020.606590)
Supplement: Supplementary file 1 [file Data_Sheet_1.pdf]

# Supplementary Material

## 1 SUPPLEMENTARY DATA

### 1.1 Distance estimation through motion and pivoting-parallax

We use the equation of the OF experienced by a moving agent we described in method from Koenderink and Van Doorn. In this equation the speed of a translating agent was combine with the nearness of the surrounding into a term called the reduced nearness or time to contact, $\mu_i$. For the purpose of this demonstration the speed of the agent,$v$ and the real nearness, $\mu$ will be now re-introduced in equation (L2). The rotation R of the agent can be written as a rotation of rate $\vec{\omega}$.

$$\begin{aligned} \tilde{\text{OF}} &= \mu_i(\vec{t} - (\vec{t} \times \vec{d_i})\vec{d_i}) - R \times \vec{d_i} && \text{L1} \\ \tilde{\text{OF}} &= -v\mu(\vec{t} - (\vec{t} \times \vec{d_i})\vec{d_i}) - \vec{\omega} \times \vec{d_i} && \text{L2} \end{aligned} \tag{S1}$$

From L2 we notice that if $\vec{\omega}$ is null, i.e. the agent is not rotating, the OF is proportional to the speed and nearness ($\mu\ v$). This is the case during pure translation, thus motion parallax. We will now describe how distance could be obtain during pivoting parallax.

The agent pivot around a certain point in space, the pivoting point, $P$. The pivot point is aligned with the agent along $d$. Consequently, the vector between the agent and $P$ can be denoted as $\vec{P} = \mu_p \vec{d}$.

We will now look for other points than $P$ being either closer or further away than $P$ but in the same direction $\vec{d}$. We consider $P'$, with a certain distance relative to $P$. The distance is expressed such as $(\mu_p - \mu)$ with $\mu$ the signed nearness between $P$ and $P'$. When $\mu_p > \mu > 0$ the point $P'$ is closer than $P$, when $\mu < 0$ is further away than $P$, to the agent. Hence we can write, $\vec{P'} = (\mu_p - \mu)\vec{d}$ in (S2).

We now have OF at $P'$ is:

$$\begin{aligned} \vec{OF}(P') &= -v(\mu_p - \mu)(\vec{u} - (\vec{u}.\vec{d})\vec{d}) - \vec{\omega} \times \vec{d} \quad \text{L1} \\ &= +v\mu(\vec{u} - (\vec{u}.\vec{d})\vec{d}) - v_p\mu(\vec{u} - (\vec{u}.\vec{d})\vec{d}) - \vec{\omega} \times \vec{d} \quad \text{L2} \\ &= +v\mu(\vec{u} - (\vec{u}.\vec{d})\vec{d}) + \vec{OF}(P) \quad \text{L3} \\ &= +v\mu(\vec{u} - (\vec{u}.\vec{d})\vec{d}) \quad \text{because } \vec{OF}(P) = \vec{0} \quad \text{L4} \end{aligned} \tag{S2}$$

We see that the optic-flow in the direction of the pivot point variate as the function of the nearness to the pivot point (S2,L4).

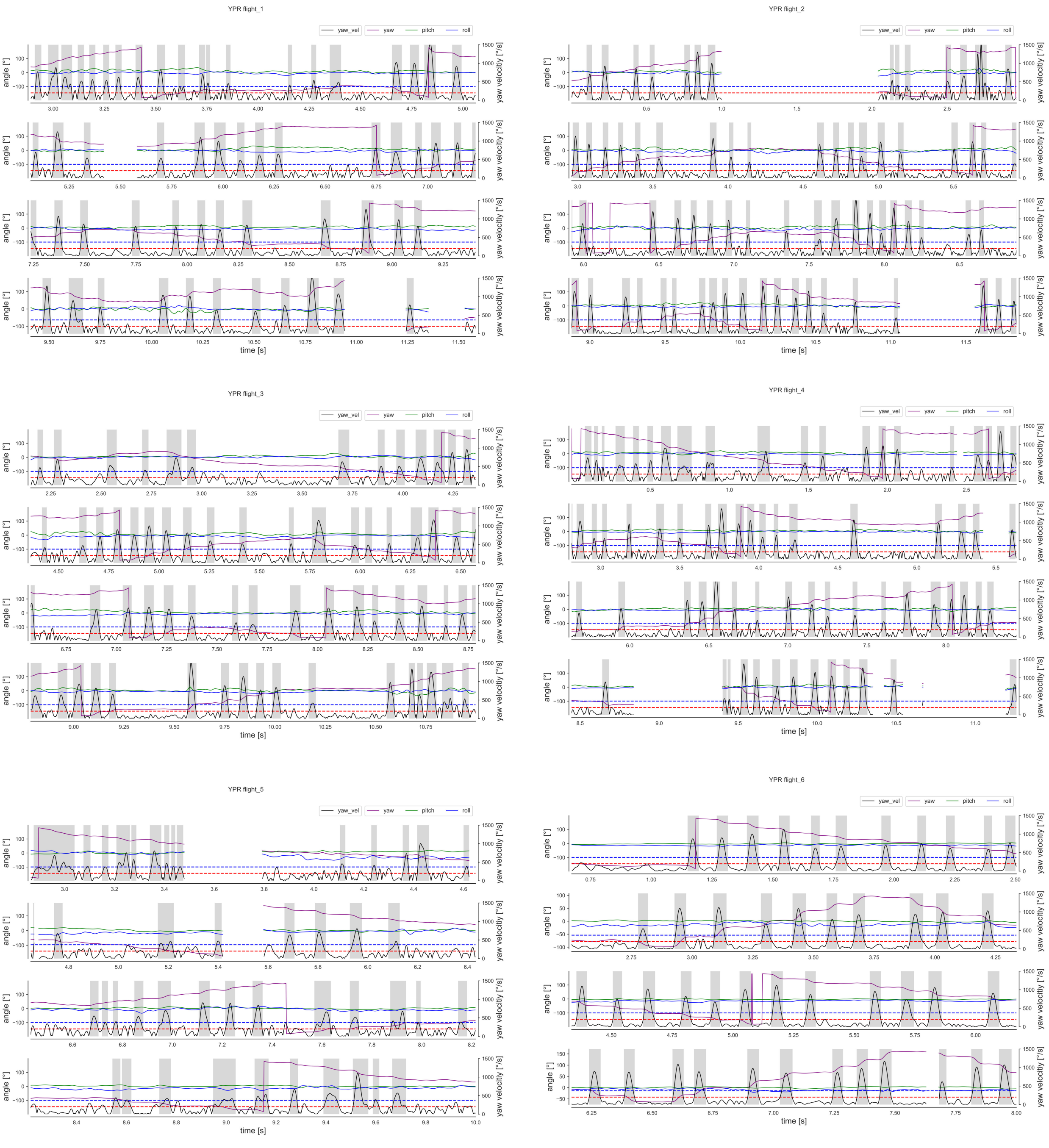


**Figure S1.** Head spatial orientation. Filtered time courses of the head YPR orientation for all recorded flights, with yaw in purple, pitch in green, roll in blue. Each orientation is overlaid with the standard deviation of the error in degrees. Rectified yaw velocity on the right axis (black). Grey shaded areas represents saccades determined by the two-thresholds method (see text): for the head, onset threshold (upper blue line) = $372.42^{\circ}.s^{-1}$ and ending threshold 2 (lower red line) = $200.5\ ^{\circ}.s^{-1}$.
