# Supplementary figures and images for: The Critical Role of Head Movements for Spatial Representation During Bumblebees Learning Flight
Source: Front Behav Neurosci. 2021 Jan 19;14:606590. doi: 10.3389/fnbeh.2020.606590 (PMC7852487; doi:10.3389/fnbeh.2020.606590)

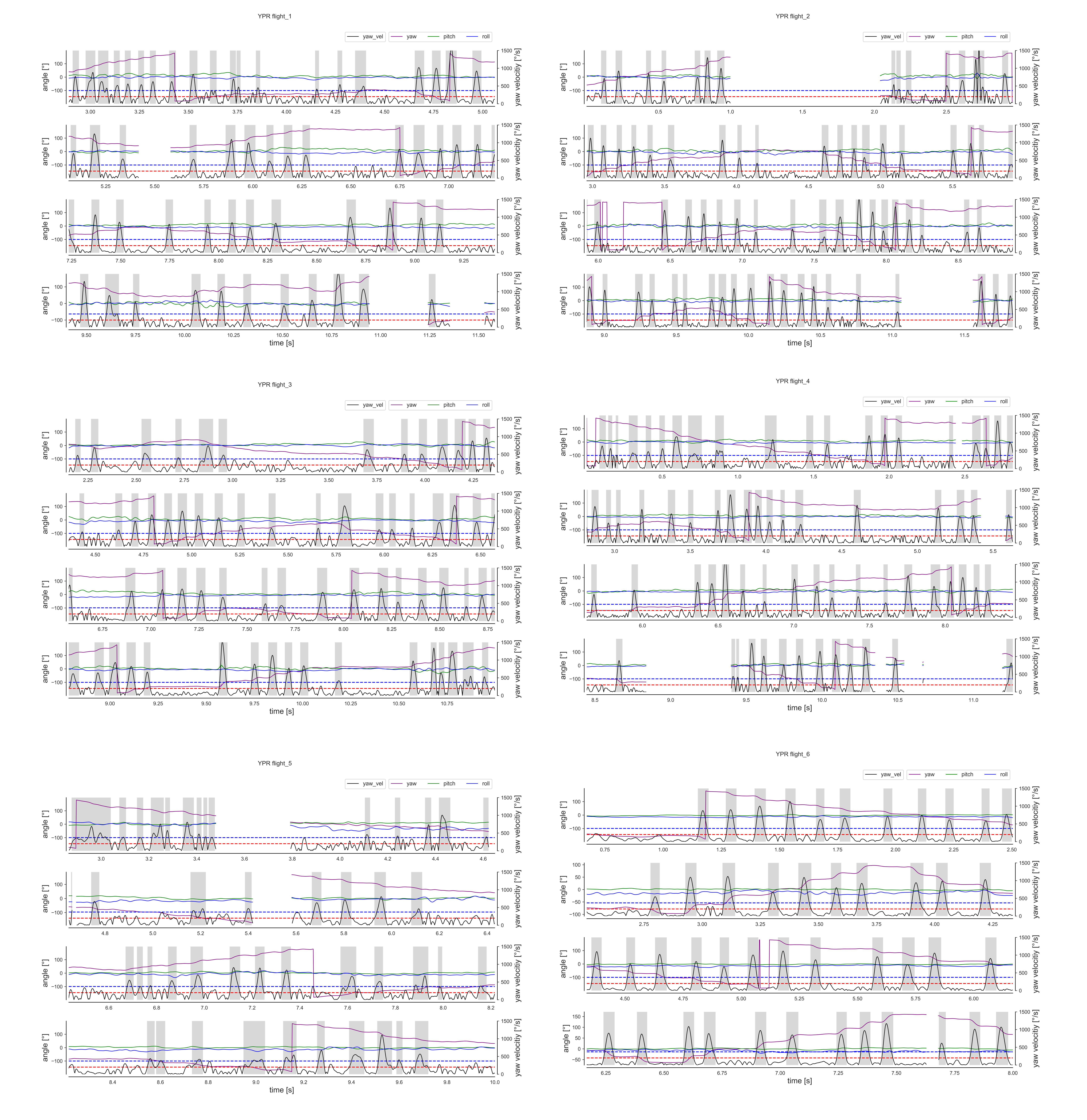

Supplement: Supplementary file 2 [file Image_1.JPEG]
